# Cellular senescence of renal tubular epithelial cells in diabetic kidney disease: emerging mechanisms and targeted interventions

**DOI:** 10.3389/fcell.2026.1973741

**Published:** 2026-09-21

**Authors:** Junwei Shi, Jianing Sun, Junwei Gao, Jun Luo, Yinzhong Dai, Shimei Hua, Chenguang Wu, Lifan Wang, Peng Liu

**Affiliations:** 1 Renal Division, Department of Medicine, Heilongjiang Academy of Chinese Medicine Sciences, Harbin, China; 2 Ningbo Municipal Hospital of Traditional Chinese Medicine, Affiliated Hospital of Zhejiang Chinese Medical University, Ningbo, China; 3 Xiyuan Hospital, China Academy of Chinese Medical Sciences, Beijing, China

**Keywords:** cellular senescence, diabetic kidney disease, renal tubular epithelial cells, senescence-associated secretory phenotype, targeted therapy

## Abstract

Diabetic kidney disease (DKD) is a leading cause of end-stage kidney disease (ESKD), and while current standard treatment strategies can slow disease progression, they are still unable to reverse progressive structural damage to the kidneys. Emerging evidence indicates that the senescence of renal tubular epithelial cells (RTECs) is not only a central feature of early-stage DKD but also a key pathological mechanism driving renal interstitial fibrosis and progressive kidney function decline. This review systematically elucidates the emerging molecular networks governing RTEC senescence within the DKD microenvironment. Specifically, it thoroughly explores the roles of glucolipotoxicity induced by metabolic dysregulation, persistent reactive oxygen species (ROS) stress, DNA damage, senescence-associated secretory phenotype (SASP)-mediated paracrine toxicity, abnormal epigenetic modifications, and failure of endogenous protective mechanisms such as mitophagy. Furthermore, this review provides a brief overview of current potential therapeutic strategies for delaying or eliminating senescent cells, covering research progress and clinical limitations related to sodium-glucose cotransporter 2 (SGLT2) inhibitors, metformin, mesenchymal stem cell (MSC) therapy, and potential natural products.

## 1 Introduction

Diabetic kidney disease (DKD), a major microvascular complication of diabetes, remains the leading global cause of chronic kidney disease (CKD) and end-stage kidney disease (ESKD) (Glastras and Pollock, 2024; Abbad et al., 2025). Which is characterized by excessive accumulation and deposition of extracellular matrix, tubular atrophy, dilatation and necrosis (Liu, 2024; De Gregorio et al., 2025; Liu L. et al., 2026) that was associated with diverse underling molecular mechanisms, such as the activation of oxidative stress and inflammation (Tang and Yiu, 2020; Rayego-Mateos et al., 2023; Wang et al., 2023a; Liu H. et al., 2025), renin-angiotensin system (Walther et al., 2021; Zhao et al., 2025) and TGF-β/Smad (Hong et al., 2025; Liu K. et al., 2025) and Wnt/β-catenin (Miao et al., 2023; Long et al., 2024; Tian et al., 2024) signaling pathway as well as the dysbiosis of gut microbiota (Stepanova, 2024; He et al., 2025; Liu et al., 2026c; Sun et al., 2026; Zhang et al., 2026) and the disorder of endogenous metabolites (Wang et al., 2023b; Corradi et al., 2024; Li et al., 2024c; Sharma et al., 2025; Song et al., 2025). Epidemiological data show that approximately 40% of patients with type 2 diabetes (T2DM) and 30% of patients with type 1 diabetes (T1DM) develop DKD, a condition that accounts for more than one-third of all cases of ESKD worldwide (Anders et al., 2018; Disease and Collaboration, 2020). Current standard treatment strategies for DKD include strict glycemic control, antihypertensive therapy, renin-angiotensin-aldosterone system (RAAS) blockers, and sodium-glucose cotransporter 2 (SGLT2) inhibitors and nonsteroidal mineralocorticoid receptor antagonists (Barrera-Chimal et al., 2022; Correa-Rotter et al., 2024). Despite these therapeutic advances, current interventions remain unable to completely halt or reverse the underlying structural kidney damage (Zhao et al., 2018; Fioretto and Pontremoli, 2022; He et al., 2024).

Historically, pathological research and clinical interventions for DKD have primarily focused on abnormal changes in glomerular hemodynamics, thickening of the basement membrane, and damage to and shedding of podocytes (Chen Y. et al., 2024; Yin et al., 2025; Zhu et al., 2026). However, a growing body of clinical evidence and basic research indicates that renal tubular epithelial cell (RTEC) injury is not only a central feature of early-stage DKD but also a key determinant of kidney function decline and long-term patient prognosis (Balakumar, 2024; Liu D. et al., 2024; Liu et al., 2025c). Under diabetic conditions, RTECs undergo profound phenotypic transitions and functional impairment. Among these changes, cellular senescence—a stress response to injury—has been established as a key pathological mechanism driving tubulointerstitial fibrosis and disease progression in DKD (Schunk et al., 2021; Chen et al., 2022) as detailed in Figure 1.

**FIGURE 1:**
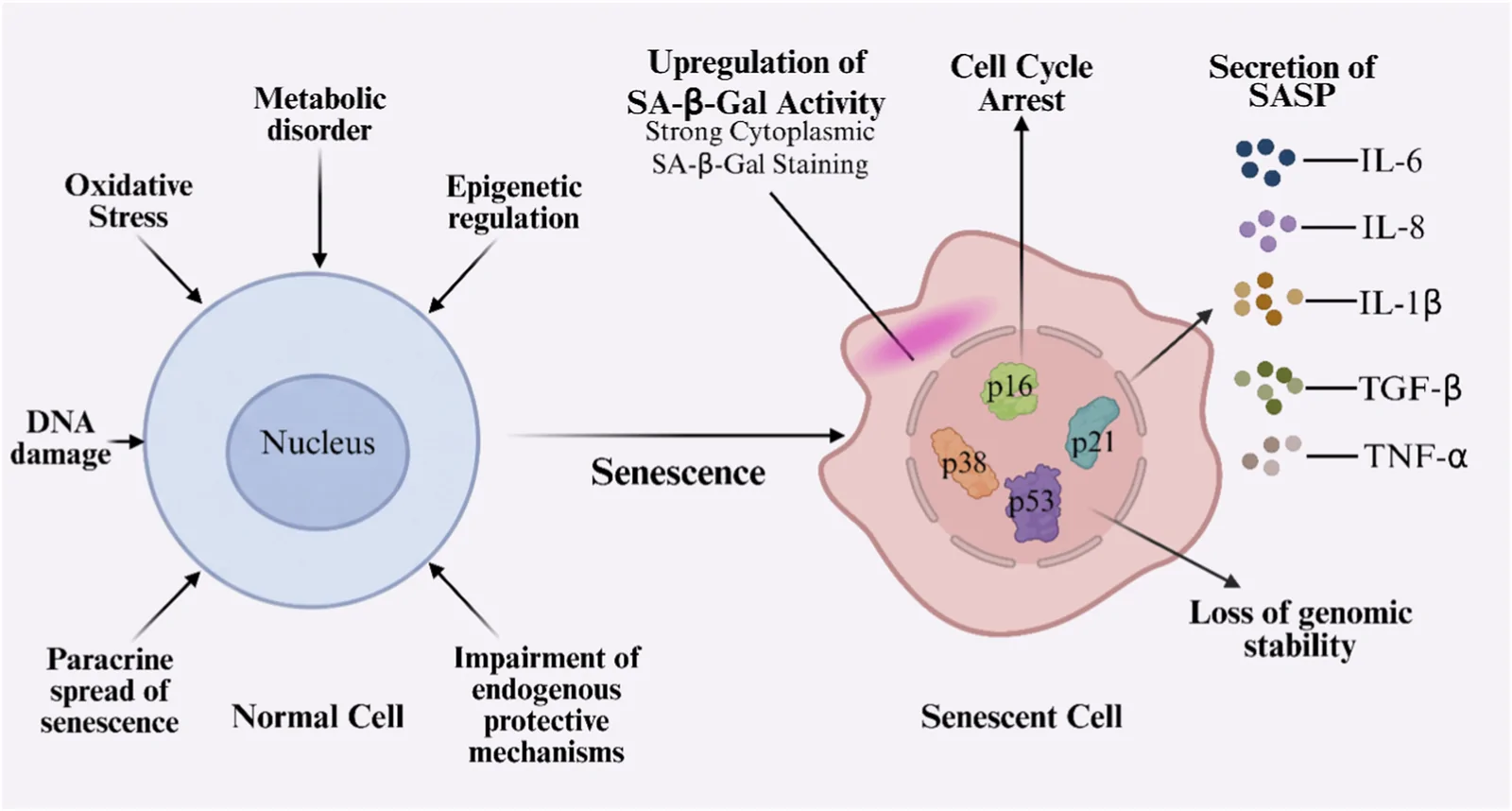
Stimuli induce cellular senescence Figure Caption:DNA damage, paracrine propagation of senescence, metabolic dysregulation, oxidative stress, epigenetic regulation, and compromised endogenous protective mechanisms collectively contribute to the induction of cellular senescence. Senescent cells are consequently marked by sustained cell cycle arrest, genomic instability, and robust SASP secretion, all of which perpetuate tissue dysfunction and promote pathological progression. Created in BioRender. s, J. (2026) https://core.local.biorender.dev/api/short-link/ofhjdpe. Note. Abbreviations: DKD, diabetic kidney disease; IL, interleukin; RTEC, renal tubular epithelial cell; SA-β-Gal, senescence-associated β-galactosidase; SASP, senescence-associated secretory phenotype; TGF-β, transforming growth factor-β; TNF-α, tumor necrosis factor-α. p16, p38, and p53 are classical signaling molecules involved in cell cycle regulation and stress responses, and their upregulated expression is closely associated with the senescent process.

Increasing publications have suggested that cellular senescence is involved in CKD (Liu et al., 2023; Li et al., 2024b; Xiang et al., 2024; Yang et al., 2024a; Wang Y. B. et al., 2025). Cellular senescence promotes the progression of DKD by contributing to pathological processes such as the amplification of inflammation, tissue remodeling, and the exacerbation of fibrosis (Wei et al., 2024; Patel et al., 2026). The accumulation of senescent RTECs impairs the intrinsic regenerative capacity of the kidney (Lu et al., 2024b; Zhong et al., 2025). More importantly, these cells—which remain in a state of cell cycle arrest but exhibit highly active metabolism—secrete senescence-associated secretory phenotype (SASP) factors (Wang W. et al., 2026). These factors induce senescence in neighboring healthy cells through paracrine effects, ultimately driving tubular atrophy and tubulointerstitial fibrosis (Li et al., 2023c). This review aims to summarize recent findings on novel mechanisms of RTEC senescence in DKD and summarize progress regarding emerging drugs in delaying RTEC senescence, with the goal of providing a new theoretical perspective for clinical interventions in DKD.

## 2 Pathological links between cellular senescence and RTECs in DKD

RTECs constitute most of the renal parenchyma and represent the most metabolically active cell population in the kidney (Qi and Yang, 2018; Cao et al., 2022; Bai et al., 2024; Wei et al., 2025). RTECs rely on ATP supplied by mitochondria to maintain high-intensity reabsorption of ions, glucose, and solutes (Li Y. et al., 2024; Deng et al., 2025; Zhou et al., 2025). Under DKD conditions, the intracellular accumulation of glucose and lipids impairs the kidneys’ normal metabolic pathways, driving metabolic reprogramming (Wang Y. et al., 2024; Li and Susztak, 2025; Cao et al., 2026). A persistently high-glucose microenvironment leads to massive glucose uptake by RTECs (Ahmad et al., 2022). This sustained hyper-reabsorption triggers a severe imbalance in energy metabolism and exacerbates intracellular hypoxia within the RTECs (Hou et al., 2024; Lu et al., 2024a).

Increasing studies indicated that metabolic reprogramming is closely related to kidney injury (Wang W. et al., 2024; Ding et al., 2025). Metabolic reprogramming in RTECs shifts cellular energy utilization from fatty acid oxidation and oxidative phosphorylation to glycolysis (Wang T. et al., 2024; Sun et al., 2025). This inefficient energy metabolism leads to insufficient ATP supply and causes unmetabolized lipids to accumulate intracellularly as lipid droplets, creating a lipotoxic environment (Cai et al., 2020; Zhao L. et al., 2023). Concurrently, lipotoxicity and advanced glycation end products (AGEs) jointly drive mitochondrial dysfunction and endoplasmic reticulum stress, triggering excessive reactive oxygen species (ROS) production and overwhelming the cellular antioxidant defense systems (Liu et al., 2025d; Wu et al., 2026). If the damage to the RTECs goes beyond the threshold for self-repair, the cells enter senescence. The most obvious feature of these senescent cells is permanent cell cycle arrest. This process depends heavily on two main signaling axes: tumor protein p53 (p53)/cyclin-dependent kinase inhibitor 1A (p21) and p16^INK4a^. Both of these ultimately feed into the retinoblastoma (Rb) pathway (Gao et al., 2022). Early on during DNA damage, the ataxia-telangiectasia mutated (ATM) kinase gets activated. This kinase then phosphorylates the p53 protein to keep it stable. As a transcription factor, the activated p53 strongly drives the expression of its downstream target, p21 (Li et al., 2025). Once produced, p21 binds to cyclin E-cyclin-dependent kinase 2 (CDK2) complexes and blocks their activity, which effectively stops the cell cycle from moving forward (Eleftheriadis et al., 2022). Later on, maintaining this senescent state over a long period requires the p16^INK4a^-CDK4/6-Rb pathway. What happens here is that p16^INK4a^ specifically inhibits CDK4/6. Because of this inhibition, the Rb protein stays in a hypophosphorylated state. In this state, Rb binds tightly to E2F transcription factors. This binding completely shuts down the transcription of genes that the cell needs to copy its DNA (Wei et al., 2024; Lu et al., 2025). Consequently, damaged or apoptotic RTECs cannot be replaced due to this permanent cell cycle exit, which directly leads to dilation of the renal tubular lumen, epithelial thinning, and tubular atrophy. This severely compromises the integrity of the renal tubular epithelial barrier and its reabsorption function, and constitutes an important histopathological basis for the progressive decline in kidney function (Al-Dabet et al., 2022). At the morphological and structural levels, senescent RTECs undergo significant changes, characterized by cellular hypertrophy and a flattened morphology (Huang et al., 2022). These changes are accompanied by nuclear structural remodeling, including a significant downregulation of lamin B1 expression and impaired nuclear membrane integrity, and chromatin remodeling into senescence-associated heterochromatin foci. Together, these changes indicate a decline in genomic stability (Lukášová et al., 2018).

The deleterious impact of senescent RTECs on kidney structure and function stems not only from their own loss of function but also from their pathological remodeling of the surrounding microenvironment, a process primarily mediated by the release of SASP (Xiong et al., 2024). The SASP is a biologically active secretome comprising various pro-inflammatory cytokines, chemokines, and profibrotic growth factors (Phillips et al., 2025). Through these secreted factors, senescent RTECs recruit monocytes and T lymphocytes to the tubulointerstitium. In the DKD microenvironment, infiltrating monocytes differentiate into macrophages and further polarize toward a profibrotic phenotype, exacerbating the deterioration of the local microenvironment (Chu et al., 2025). Furthermore, SASP mediators induce a partial epithelial-to-mesenchymal transition (pEMT) in adjacent RTECs. Key effector molecules, including the profibrotic cytokine transforming growth factor-β (TGF-β), the pro-inflammatory interleukins interleukin-6 (IL-6), interleukin-1β (IL-1β), and interleukin-8 (IL-8), tumor necrosis factor-α (TNF-α), as well as the matrix-remodeling protease matrix metalloproteinase-9 (MMP-9), have been identified as principal drivers of this process (Yang et al., 2023; Lin et al., 2024). These factors synergistically activate intracellular signaling cascades that exacerbate the diffuse progression of tubulointerstitial fibrosis. In addition, the SASP exerts a paracrine senescence effect; the ROS and inflammatory cytokines it releases can induce damage in neighboring healthy cells and drive them into a senescent state. This cycle leads to the focal accumulation of senescent cells within kidney tissue and ultimately accelerates widespread functional loss of nephrons (Wang C. et al., 2025).

## 3 Mechanisms underlying RTEC senescence

In recent years, research into the pathogenesis of DKD has made significant progress, revealing multiple molecular networks that drive the senescence of RTECs (Zhang et al., 2025a; Hu et al., 2026b) as detailed in Table 1 and Figure 2. This has provided deeper mechanistic insights into the complexity of senescence in DKD and highlights novel targets for precision interventions (Chen et al., 2018; Zhang et al., 2025b).

**TABLE 1:** Mechanisms and treatments that regulate renal tubular epithelial cell senescence in diabetic kidney disease.

Experimental model	Signaling pathway	Effect	Treatment	References
C57BL/6 mice HK-2 cells	PA→RXRα↑/MR → CDKN1A (p21)↑ →Cellular Senescence	Cellular senescence Lipid accumulation Increased SASP factors	MR antagonist	(Feng et al., 2024)
*db/db* mice NRK-52 E cells	ALM→RXRα↑→ CDX2↑→ CFTR ↑ → β-catenin↓→ Snail↓→ EMT	Tubulointerstitial fibrosis	α-Lipoamide (ALM)	(Zhang et al., 2023)
*ApoE* ^−/−^ mice GEnCs	HG→RXRα↓→ circ8411↓→ miR-23a-5p↑ →ABCA1↓ →Pyroptosis	Pyroptosis	GLP-1RA	(Wu et al., 2024)
*Hhip* PTC-Tg mice HK-2 cells	HG→ATM/ATR↑→CDK1/CDK2↓→Cellular Senescence	Cellular senescence Glucotoxicity Increased SASP factors	Canagliflozin	(Zhao et al., 2023b)
WT C57BL/6 mice HKC-8 cells	HG→FXII ↑→uPAR→ FAK/Src↑→NOX1↑→ROS↑→ DNAdamage→↑ATM/ATR↑→p53/p21↑ →Cellular Senescence	Cellular senescence Excessive ROS Increased SASP factors	FXII blockade	(Elwakiel et al., 2024)
*eNOS* ^−/−^ *db/db* mice hGECs hPTECs	DKD→ROS↑→ASK-1↑→p38 MAPK → p53/p21→Cellular Senescence	Cellular senescence Increased SASP factors	Selective ASK-1 inhibitor	(Kajimoto et al., 2024)
*Akita* mice *Agt* PT-KO mice HK-2 cells MDCK cells	DKD→iRAS↑→Ang II↑→NOX4↑→ROS↑→p16 →Cellular Senescence	Cellular senescence Increased SASP factors	Targeted *Agt* knockout Losartan N-acetylcysteine	(Yang et al., 2026)
*db/db* mice NRK-52 E cells NRK-49 F cells	Senescent cells→Shh↑ →NRK-49 F→Tubulointerstitial fibrosis	Tubulointerstitial fibrosis	Cyclopamine	(Wang et al., 2022)
HK-2	HG+TGFβ1→Serpin E1↑ →Cellular Senescence	Cellular senescence	Serpin E1 inhibitor Dasatinib + Quercetin (D+Q)	(Chen et al., 2023)
C57BL/6 J mice *Glis1* KO/Tg mice mTECs	HG→Glis1↓→KAT5↑→Histone lactylation→Cellular Senescence	Cellular senescence Increased SASP factors	*Glis1* overexpression, Sodium	(Chen et al., 2025b)
*db/db* mice STZ-induced mice *NAT10* KO mice HK-2 cells	DKD→NAT10↑→ac4C acetylation of *PAPP-A* mRNA→PAPP-A↑ →p53↑→Cellular Senescence	Cellular senescence	Targeted *NAT10* knockout AAV Remodelin	(Hu et al., 2026b)
HK-2 cells	HG→ER-α36↓→EZH2↓→H3K27me3↓→PTEN↑ →PI3K/AKT↓→Cellular Senescence	Cellular senescence	ER-α36 overexpression	(Yu et al., 2025)
C57BL/6 mice *YME1L* Tg mice HK-2 cells	DKD→YME1L↓→BCL2L13/LC3↓→Mitophagy inhibition→mtROS↑ →Cellular Senescence	Cellular senescence Increased SASP factors	*YME1L* overexpression	(Luo et al., 2024)
C57BL/6 J mice *ERRα* ptKO/ptKI mice HK-2 cells	DKD→ERRα↓→NOS2↓→Citrulline↓ →ROS →Cellular Senescence	Cellular senescence Mitochondrial dysfunction Increased SASP factors	Citrulline	(Hu et al., 2026a)
C57BL/6 mice RTECs	HG→Parkin↓→GATA4↑→GAS1↑ →Cellular Senescence	Cellular senescence Increased SASP factors	Parkin overexpression	(Chen et al., 2020)

AAV, adeno-associated virus; ABCA1, ATP-binding cassette subfamily A member 1; ac4C, N4-acetylcytidine; Agt, angiotensinogen (gene); AKT, protein kinase B; ALM, α-lipoamide; Ang II, angiotensin II; ApoE, apolipoprotein E; ASK-1, apoptosis signal-regulating kinase 1; ATM, ataxia telangiectasia mutated; ATR, ATM, and Rad3-related; BCL2L13, Bcl-2-like protein 13; CDK1, cyclin-dependent kinase 1; CDK2, cyclin-dependent kinase 2; CDKN1A, cyclin-dependent kinase inhibitor 1A (p21); CDKN2A, cyclin-dependent kinase inhibitor 2A (p16); CDX2, caudal type homeobox 2; CFTR, cystic fibrosis transmembrane conductance regulator; circ8411, circular RNA 8411; D+Q, dasatinib plus quercetin; DKD, diabetic kidney disease; DSBs, DNA double-strand breaks (referred to as DNA damage in the table); EMT, epithelial-to-mesenchymal transition; eNOS, endothelial nitric oxide synthase; ER-α36, estrogen receptor alpha 36; ERRα, estrogen-related receptor alpha; EZH2, enhancer of zeste homolog 2; FAK, focal adhesion kinase; FXII, coagulation factor XII; GAS1, growth arrest-specific 1; GATA4, GATA binding protein 4; GEnCs, glomerular endothelial cells; Glis1, GLI-similar 1; GLP-1RA, glucagon-like peptide-1 receptor agonist; H3K27me3, trimethylation of histone H3 lysine 27; HG, high glucose; hGECs, human glomerular endothelial cells; hPTECs, human proximal tubular epithelial cells; HKC-8, human kidney cell line-8; HK-2, human kidney 2; iRAS, intrarenal renin-angiotensin system; KAT5, lysine acetyltransferase 5; KO, knockout; LC3, microtubule-associated protein 1 light chain 3; MDCK, Madin-Darby canine kidney; miR-23a-5p, microRNA-23a-5p; MR, mineralocorticoid receptor; mTECs, mouse tubular epithelial cells; mtROS, mitochondrial reactive oxygen species; NAT10, N-acetyltransferase 10; NOS2, nitric oxide synthase 2; NOX1, NADPH oxidase 1; NOX4, NADPH oxidase 4; NRK-49F, normal rat kidney 49F; NRK-52E, normal rat kidney 52E; p38 MAPK, p38 mitogen-activated protein kinase; PA, palmitic acid; PAPP-A, pregnancy-associated plasma protein A; PI3K, phosphoinositide 3-kinase; PT, proximal tubule; PTEN, phosphatase and tensin homolog; ROS, reactive oxygen species; RTECs, renal tubular epithelial cells; RXRα, retinoid X receptor alpha; SASP, senescence-associated secretory phenotype; Serpin E1, serpin family E member 1; Shh, Sonic Hedgehog; Src, proto-oncogene tyrosine-protein kinase Src; STZ, streptozotocin; TGFβ1, transforming growth factor beta 1; Tg, transgenic; uPAR, urokinase-type plasminogen activator receptor; WT, wild type; YME1L, YME1-like 1 ATPase. Arrows (↑ and ↓) indicate upregulation and downregulation, respectively.

**FIGURE 2:**
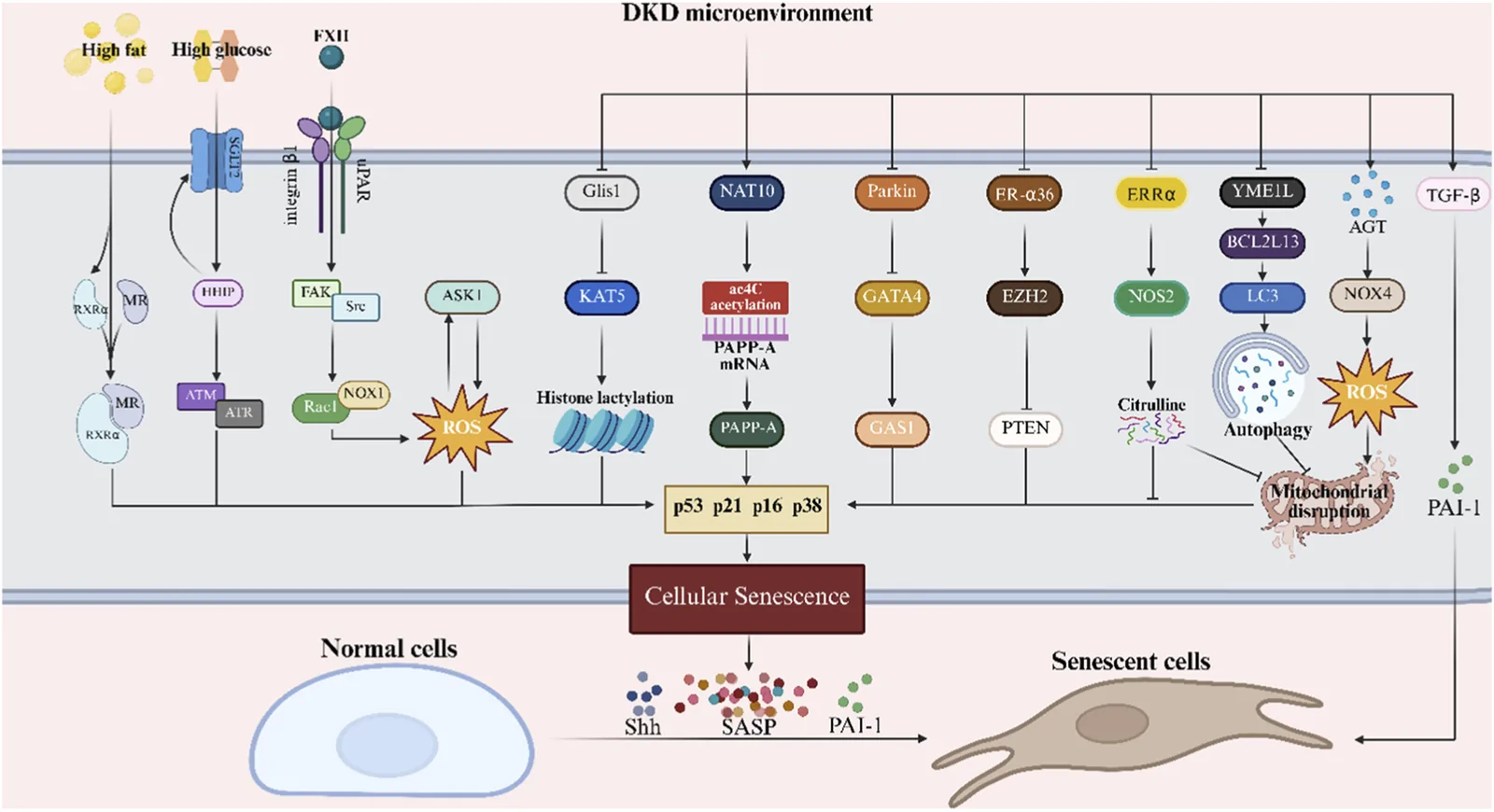
Emerging mechanisms of renal tubular epithelial cell senescence in diabetic kidney disease. Figure Caption:This schematic diagram comprehensively illustrates the complex regulatory network underlying cellular senescence induced by the diabetic kidney disease (DKD) microenvironment, which is orchestrated by multiple parallel intracellular signaling pathways. Specifically, these upstream pathways encompass RXRa/MR, SGLT2-HHIP-ATM/ATR, FXII-mediated activation of FAK/Src leading to Rac1/NOX1-driven ROS production, the ASK1/ROS axis, Glis1-KAT5-mediated histone lactylation, NAT10-mediated ac4C acetylation of PAPP-A mRNA, the Parkin-GATA4-GAS1 axis, the ER-α36-EZH2-PTEN axis, the ERRα-NOS2 axis, YME1L-BCL2L13-mediated autophagy-associated mitochondrial disruption, as well as oxidative stress responses involving AGT-NOX4 and TGF-β-PAI-1. Collectively, these diverse signaling events converge to activate the core senescence proteins p53, p21, p16, and p38, ultimately promoting the phenotypic transition of normal cells into senescent cells characterized by a flattened morphology. Subsequently, these senescent cells secrete senescence-associated factors, including Shh, SASP, and PAI-1, which act in a paracrine manner on surrounding normal cells, thereby establishing a vicious positive feedback loop that accelerates DKD pathological progression. Created in BioRender. s, J. (2026) https://core.local.biorender.dev/api/short-link/06emrgm. Note. Abbreviations: AGT, Angiotensinogen; ac4C, N4-acetylcytidine; ASK1, Apoptosis signal-regulating kinase 1; ATM, Ataxia telangiectasia mutated; ATR, ATM and Rad3 related; BCL2L13, BCL2-like 13; DKD, Diabetic Kidney Disease; ER-α36, Estrogen receptor alpha 36; ERRα, Estrogen-related receptor alpha; EZH2, Enhancer of zeste homolog 2; FAK, Focal adhesion kinase; FXII, Coagulation factor XII; GAS1, Growth arrest specific 1; GATA4, GATA binding protein 4; Glis1, GLI-similar 1; HHIP, Hedgehog interacting protein; KAT5, Lysine acetyltransferase 5; LC3, Microtubule-associated protein 1A/1 B-light chain 3; MR, Mineralocorticoid receptor; mRNA, Messenger RNA; NAT10, N-acetyltransferase 10; NOS2, Nitric oxide synthase 2; NOX1, NADPH oxidase 1; NOX4, NADPH oxidase 4; p16, Cyclin-dependent kinase inhibitor 2A; p21, Cyclin-dependent kinase inhibitor 1A; p38, p38 mitogen-activated protein kinase; p53, Tumor protein 53; PAI-1, Plasminogen activator inhibitor-1; PAPP-A, Pregnancy-associated plasma protein-A; Parkin, E3 ubiquitin-protein ligase Parkin; PTEN, Phosphatase and tensin homolog; Rac1, Ras-related C3 botulinum toxin substrate 1; ROS, Reactive oxygen species; RXRa, Retinoid X receptor alpha; SASP, Senescence-associated secretory phenotype; SGLT2, Sodium-glucose cotransporter 2; Shh, Sonic hedgehog; Src, Proto-oncogene tyrosine-protein kinase Src; TGF-β, Transforming growth factor beta; uPAR, Urokinase-type plasminogen activator receptor; YME1L, YME1-like 1.

### 3.1 Metabolic dysregulation driving senescence

RTEC senescence is closely associated with the metabolic dysregulation in DKD. Feng et al. demonstrated that in the high-fat diet/streptozotocin (HFD/STZ)-induced DKD model, the expression of retinoid X receptor alpha (RXRα) was significantly elevated in RTECs. In this context, RXRα directly binds to the mineralocorticoid receptor (MR) to synergistically activate the transcription of downstream senescence-related genes, such as p21, thereby driving cellular senescence. Furthermore, the RXRα/MR complex can also disrupt renal glucose and lipid metabolism by upregulating phosphoenolpyruvate carboxykinase 1 (PCK1) and pyruvate dehydrogenase kinase 4 (PDK4), further exacerbating the senescence cycle (Feng et al., 2024). However, recent studies indicate that α-lipoamide can delay DKD fibrosis by upregulating RXRα and activating the RXRα/caudal type homeobox 2 (CDX2)/cystic fibrosis transmembrane conductance regulator (CFTR)/β-catenin signaling pathway (Zhang et al., 2023). Additionally, GLP-1 receptor agonists (GLP-1RA) can also effectively reduce cholesterol accumulation and pyroptosis by targeting the RXRα/circ8411/miR-23a-5p/ATP-binding cassette subfamily A member 1 (ABCA1) axis (Wu et al., 2024). These findings suggest that while RXRα, in concert with MR, promotes senescence and fibrosis, it can also exert pleiotropic renoprotective effects under specific pharmacological interventions. Zhao *et al.* found that overexpression of Hedgehog-interacting proteins (HHIPs) in renal proximal tubular cells (RPTCs) enhances SGLT2 protein expression and glucose uptake. This excessive influx triggers cellular glucotoxicity, activates ATM/ATM- and Rad3-related (ATR)-mediated cell cycle arrest, and induces cellular senescence—a pathogenic cascade that can be effectively abrogated by the SGLT2 inhibitor canagliflozin (Zhao X. P. et al., 2023). Both the RXRα/MR and HHIP pathways are activated by DKD-induced metabolic dysregulation. By reprogramming intracellular metabolic pathways, they exacerbate glucolipotoxicity, ultimately culminating in RTEC senescence and tubulointerstitial fibrosis.

### 3.2 ROS and DNA damage driving senescence

Persistent oxidative stress and DNA damage are pivotal drivers of cellular senescence in RTECs (Small et al., 2012; Wang et al., 2019; Kim et al., 2026). Coagulation factor XII (FXII) is a serine protease precursor synthesized in the liver and secreted into plasma (Pham et al., 2012; Maas and Renné, 2018); it plays a key role in contact activation within the intrinsic coagulation pathway (Göbel et al., 2018; Kluge et al., 2022; Shamanaev et al., 2023). Previous studies have found that FXII levels are abnormally elevated in DKD, suggesting a possible association with inflammatory mechanisms (Xiao et al., 2023). Mechanistic studies by Elwakiel et al. have demonstrated that under pathological conditions, the proenzyme form of FXII binds to the urokinase-type plasminogen activator receptor (uPAR) on the RTEC surface and, by recruiting integrin β1 as a co-receptor, activates the focal adhesion kinase (FAK)/proto-oncogene tyrosine-protein kinase Src (Src)/Ras-related C3 botulinum toxin substrate 1 (Rac1)/NADPH oxidase 1 (NOX1) pathway, generating large amounts of ROS, which leads to persistent DNA double-strand breaks (DSBs) and subsequently triggers a p21-dependent senescence program (Elwakiel et al., 2024). Acting as a critical intracellular sensor for ROS, the apoptosis signal-regulating kinase 1 (ASK-1) pathway is deeply implicated in renal fibrosis via the upstream regulation of p38 mitogen-activated protein kinase (p38 MAPK) and c-Jun N-terminal kinase (JNK) (Tesch et al., 2016). In the DKD milieu, ROS accumulation leads to the hyperactivation of ASK-1 and its downstream p38 MAPK pathway (Liles et al., 2018). Research by Kajimoto *et al.* found that sustained activation of the p38 MAPK pathway is closely associated with the activation of the p53/p21 pathway and drives the expression of SASP, jointly promoting the entry of RTECs into a state of senescence (Kajimoto et al., 2024). Yang et al. found that high expression of angiotensinogen (AGT) specific to the proximal tubule significantly upregulates NADPH oxidase 4 (NOX4) levels (Yang et al., 2026). NOX4 is widely localized on the membranes of mitochondria and the endoplasmic reticulum; its pathological activation leads to the production of large amounts of ROS, triggering severe mitochondrial oxidative stress (Jia et al., 2024; Lai et al., 2024; Dai et al., 2026). Experimental animals exhibited disruption of mitochondrial cristae structure accompanied by oxidative DNA damage, ultimately leading to p16^INK4a^-dependent senescence and apoptosis in RTECs (Yang et al., 2026). Collectively, pathogenic mediators such as FXII and AGT induce profound ROS generation and subsequent DSBs, forcing RTECs into senescence. Concurrently, stress sensors like ASK1 amplify these oxidative signals to solidify the senescent phenotype. Thus, dismantling this ROS-senescence axis presents a therapeutic avenue for DKD intervention.

### 3.3 Paracrine signaling driving senescence

Paracrine signaling is a pivotal mechanism driving the propagation of senescence within the tubulointerstitium (Zhao et al., 2019; Hickson et al., 2021; Wang et al., 2024b). Through the secretion of SASP factors, senescent RTECs induce paracrine effects, precipitating premature senescence in adjacent, otherwise healthy cells (Yang et al., 2024b). Research by Wang *et al.* revealed that senescent RTECs can activate adjacent fibroblasts by secreting the Sonic Hedgehog (Shh) protein, promoting their proliferation and activation, thereby accelerating tubulointerstitial fibrosis (Wang et al., 2022). The TGF-β/Smads pathway is a canonical signaling cascade involved in cellular senescence. TGF-β activates Smad proteins to upregulate p21 and promote nuclear translocation of p16^INK4a^, thereby inducing cell cycle arrest in glomerular endothelial cells (Ueda et al., 2021). In RTECs, TGF-β1 induces upregulation of Serpin E1 (PAI-1). PAI-1 secreted by senescent tubular cells serves as a critical effector molecule mediating paracrine senescence, and its specific inhibitor, TM5441, effectively abrogates this paracrine effect (Chen et al., 2023). Furthermore, Smad3 downstream of TGF-β1 also contributes to DKD renal injury by suppressing autophagy in RTECs (Yang et al., 2021). However, Chen et al. found that complete blockade of interleukin-33 (IL-33) signaling paradoxically aggravated kidney injury in DKD, while partial blockade effectively ameliorated renal damage and cellular senescence (Chen L. et al., 2024). This suggests that, within a certain physiological range, some SASP factors may exert protective effects in the kidney. Nevertheless, mounting evidence indicates that their net effect in the DKD microenvironment is predominantly detrimental.

### 3.4 Epigenetic regulation accelerating senescence

A major clinical challenge in halting the progression of DKD is “metabolic memory,” in which kidney damage persists even after blood glucose levels have been brought under control (Kato and Natarajan, 2019; Chen Z. et al., 2024). Research evidence suggests that metabolic memory is primarily mediated by epigenetic mechanisms, including histone modifications, DNA methylation, and non-coding RNA dysregulation (Kato and Natarajan, 2019).

Chen *et al.* demonstrated that in patients with DKD and in an STZ-induced DKD mouse model, the transcription factor GLI-similar 1 (Glis1) was significantly downregulated in RTECs. Glis1 can directly bind to the lysine acetyltransferase 5 (KAT5), interfering with its interaction with histones, thereby reducing the level of lactylation and delaying cellular senescence. Overexpression of Glis1 can alleviate high-glucose-induced senescence of RTECs and renal fibrosis (Chen et al., 2025b). Hu *et al.* revealed that N-acetyltransferase 10 (NAT10) is significantly upregulated in diabetic renal tubules, where it enhances the mRNA stability of pregnancy-associated plasma protein A (PAPP-A) via N4-acetylcytidine (ac4C) acetylation. The subsequent upregulation of PAPP-A protein interacts with p53, activating the p53/p21 signaling cascade to precipitate RTEC senescence (Hu et al., 2026b). In addition, the protective role of estrogen receptor α36 (ER-α36) in DKD, finding that ER-α36 expression is downregulated under high-glucose conditions, leading to suppressed enhancer of zeste homolog 2 (EZH2) expression, which in turn reduces the level of trimethylation of histone H3 lysine 27 (H3K27me3) modification in the phosphatase and tensin homolog (PTEN) promoter region, relieve the transcriptional repression of PTEN, upregulates PTEN, and inhibits the phosphoinositide 3-kinase (PI3K)/protein kinase B (AKT) pathway, ultimately promoting senescence and apoptosis in RTECs (Yu et al., 2025). The above studies indicate that various epigenetic modifications—including histone lactylation, RNA acetylation, and histone methylation—play important regulatory roles in the senescence of RTECs in DKD, providing a new perspective for gaining a deeper understanding of the pathogenesis of DKD and exploring therapeutic strategies targeting epigenetic regulation.

### 3.5 Impairment of endogenous protective mechanisms promoting senescence

Intrinsic protective mechanisms can regulate the anti-senescence network in multiple ways. In terms of maintaining mitochondrial quality control, the YME1-like 1 ATPase (YME1L), a mitochondrial inner membrane metalloproteinase, binds to its interacting protein BCL2 like 13 (BCL2L13) and promotes its phosphorylation. This post-translational modification enhances the interaction between BCL2L13 and microtubule-associated protein 1 light chain 3 (LC3), thereby driving the mitophagic clearance of damaged mitochondria. Consequently, this pathway mitigates mitochondrial dysfunction and excessive ROS production, effectively alleviating RTEC senescence (Luo et al., 2024). The estrogen-related receptor alpha (ERRα) acts at the transcriptional level to regulate citrulline metabolism by activating nitric oxide synthase 2 (NOS2); this metabolic axis prevents cellular senescence by maintaining mitochondrial function and redox balance (Hu et al., 2026a). Furthermore, the E3 ubiquitin ligase Parkin can target GATA binding protein 4 (GATA4), a key transcription factor involved in senescence, and by promoting its ubiquitination and degradation, effectively suppresses cell cycle arrest, inflammation, and the secretion of fibrogenic factors driven by the GATA4/growth arrest-specific gene 1 (GAS1) signaling axis (Chen et al., 2020).

## 4 Current anti-senescence strategies and limitations

### 4.1 Clinical and preclinical therapies

Senomorphics are a class of drugs that do not eliminate senescent cells but rather suppress or modulate their detrimental functions, and include agents such as SGLT2 inhibitors and metformin (Scisciola et al., 2023). Dapagliflozin prevents high-glucose-induced senescence of RTECs by blocking the C1q/TNF-related protein 1 (CTRP1)/SGLT2 axis and the ROS–DNA damage–p53/p21 signaling pathway (Eleftheriadis et al., 2022; Gu et al., 2026). Empagliflozin attenuates senescence in RTECs by upregulating sine oculis homeobox homolog 1 (Six1) and wingless-type MMTV integration site family member 4 (Wnt4), thereby suppressing nuclear factor κB (NF-κB) and its downstream inflammatory cytokines (Chen et al., 2025a). Metformin downregulates the cell cycle-related protein E2F transcription factor 1 (E2F1) in RTECs under hyperglycemic conditions, reduces the phosphorylation levels of ATM and checkpoint kinase 2 (Chk2), and consequently attenuates DNA damage and the senescent phenotype (Liang et al., 2022). Metformin upregulates the RNA-binding protein muscleblind-like protein 1 (MBNL1) and prolongs the half-life of miR-130a-3p; both of these molecules specifically bind to and inhibit the transcriptional activator signal transducer and activator of transcription 3 (STAT3), thereby significantly attenuating the accumulation of senescence-associated protein p21 and substantially reducing the proportion of senescence-associated β-galactosidase (SA-β-gal)-positive cells (Jiang et al., 2020). However, senomorphics still lack senescence-specific molecular targets, which limits their capacity to achieve precise, tissue-targeted senolysis.

Senolytics are a class of drugs that selectively induce apoptosis in senescent cells, with the core strategy being the elimination of pre-existing senescent cells (Robbins et al., 2021). Dasatinib plus Quercetin (D+Q) is currently the most extensively validated senolytic combination in DKD research, with multiple studies demonstrating its renoprotective effects. D+Q treatment not only reduces the abundance of p16^INK4a^- and p21-positive cells in the kidney, but also effectively suppresses proinflammatory macrophage infiltration and senescence-associated inflammation, while restoring the expression of the endogenous anti-aging proteins α-Klotho and Sirtuin-1 (SIRT1) (Bian et al., 2026). Alvespimycin is a potent inhibitor of heat shock protein 90 (HSP90). In mouse models of diabetes combined with acute kidney injury, short-term administration of Alvespimycin was shown to partially clear senescent RTECs, but exerted only limited improvement in renal function (Tesch et al., 2024). B-cell lymphoma 2 (Bcl-2) family inhibitors, such as navitoclax (ABT-263), attenuate sustained RTEC senescence induced by Repeated Low-Dose Cisplatin through triggering apoptotic pathways (Li S. et al., 2023). However, their efficacy in DKD remains unclear, and they carry a risk of thrombocytopenia.

Mesenchymal stem cells (MSCs) are a population of stromal cells endowed with self-renewal capacity and multipotent differentiation potential, which can be isolated from various tissues including umbilical cord, adipose tissue, and bone marrow (Han et al., 2025). They exert their therapeutic effects predominantly through the release of paracrine factors. The secretome of these cells comprises both soluble factors, such as growth factors, cytokines, chemokines, and hormones, and insoluble factors encapsulated within extracellular vesicles (EVs) (Nikfarjam et al., 2020; Trigo et al., 2025). Recent studies have revealed that MSCs and their EVs possess considerable therapeutic promise for DKD, as evidenced in both *in vitro* and *in vivo* models (Ni et al., 2023; Li Q. et al., 2024; Wang et al., 2024a; Zhang et al., 2024). Liao et al. found that MSC-derived exosomes significantly suppressed the transcription of the cell cycle negative regulators p16^INK4a^ and cyclin-dependent kinase inhibitor 2D (p19^INK4d^), while partially restoring the expression level of lamin B1. Moreover, the mRNA levels of the SASP key inflammatory factors IL-6 and C-C motif chemokine ligand 7 (CCL7) were also markedly downregulated, collectively alleviating the senescence phenotype of RTECs (Liao et al., 2021). Ko et al. demonstrated that SMUP-Cells suppressed the expression of Gremlin 2 (GREM2) in RTECs, thereby blocking Smad3 signaling and concurrently inhibiting both epithelial-to-mesenchymal transition and cellular senescence in RTECs (Ko et al., 2025). Konari et al. demonstrated that intravenously administered bone marrow-derived mesenchymal stem cells (BM-MSCs) could transfer their mitochondria to renal proximal tubular epithelial cells (PTECs) in streptozotocin-induced diabetic animals. This mitochondrial transfer attenuated PTECs injury by upregulating the expression of superoxide dismutase 2 (SOD2), the anti-apoptotic protein Bcl-2, downregulating the pro-apoptotic protein Bcl-2-associated X protein (Bax), and suppressing the production of ROS (Konari et al., 2019). In addition, the two studies primarily focusing on glomerular cells also observed the therapeutic effects of mesenchymal stem cells on RTECs. Human umbilical cord-derived MSCs (hucMSCs) activated AMP-activated protein kinase (AMPK)/mammalian target of rapamycin (mTOR) pathway-dependent autophagy through paracrine action, alleviated high glucose-induced podocyte senescence, and concurrently mitigated pathological damage to RTECs (Li et al., 2023b). Placenta-derived MSCs (P-MSCs) upregulated SIRT1/forkhead box O1 (FOXO1) pathway-mediated autophagy, significantly reduced blood glucose, blood lipids, and the urinary albumin-to-creatinine ratio in DKD rats, and ameliorated both tubular injury and glomerular mesangial matrix deposition (Liu H. et al., 2024).

Proteolysis-targeting chimeras (PROTACs) represent a novel class of small-molecule drugs composed of a ligand that recognizes the protein of interest (POI), a linker, and a ligand that engages an E3 ubiquitin ligase (He et al., 2022). PROTACs recruit specific E3 ligases via the ligand moiety to induce ubiquitination of the target protein, which is subsequently degraded through the ubiquitin-proteasome pathway (Békés et al., 2022). As diverse pathogenic proteins can be eliminated by recruiting distinct E3 ligases using tailored ligands, PROTAC technology holds considerable promise for precision drug discovery and development. For instance, P1705434, a Von Hippel-Lindau (VHL)-recruiting PROTAC, has been shown to hijack the VHL E3 ligase to promote ubiquitin-proteasome degradation of Smad3 while concomitantly attenuating VHL-mediated degradation of HIF-2α, thereby correcting the overactivation of TGF-β/Smad3 signaling and the imbalance between hypoxia-inducible factor-1α (HIF-1α) and hypoxia-inducible factor-2α (HIF-2α). This dual modulation effectively ameliorated tubular cell injury and fibrosis in both cisplatin-induced and folic acid-induced nephropathy models (Ruan et al., 2026). Additionally, the bromodomain-containing protein 4 (BRD4)-targeting PROTAC degrader AT1 has been reported to improve fibrosis in DKD by degrading BRD4 and downregulating the expression of TGF-β and IL-6 (Yang et al., 2025).

Despite their promise, clinical translation of MSCs, MSC-EVs, and PROTACs is hampered by major limitations. MSC therapies face immunogenicity, thrombosis, and tumorigenicity risks (Mei et al., 2024). MSC-EVs lack renal tropism, show only marginal kidney accumulation under pathological conditions, and have poor targeting efficiency, with long-term safety and efficacy unverified. PROTACs suffer from off-target effects, E3-ligase-dependent non-specific degradation, and poor pharmacokinetics. Moreover, clinical evidence for these approaches in DKD remains absent.

### 4.2 Natural products with anti-senescence potential

Increasing clinical evidence has highlighted that traditional Chinese medicines (TCM) were demonstrated to be as a key therapy for treating CKD patients (Mao et al., 2020; Dong et al., 2024; Acquah-Mills et al., 2025; Chen Y. et al., 2025; Wu et al., 2025; Wang K. E. et al., 2026). Accumulating studies have suggested that CKD, including DKD, could be improved by TCM prescriptions (Ruan et al., 2024; Amarasiri et al., 2025; Yuan et al., 2025; Liu et al., 2026b; Liu et al., 2026d), medicinal plant extracts (Liu P. Y. et al., 2024; Gao et al., 2025; Jiang et al., 2025; Miao et al., 2025) and natural compounds (Miao et al., 2022; Ajibade et al., 2024; Li et al., 2024d; Pan et al., 2024; Wang Y. N. et al., 2024; Ben-Azu et al., 2025; Miao and Vaziri, 2025). Many natural products, including TCM, also show some anti-senescence potential (Zhang et al., 2022; Teh et al., 2024; Wang Y. et al., 2025; Yan et al., 2025a; Yan et al., 2025b; Qiao et al., 2026). Quercetin, a natural flavonoid, can, in addition to its combined use with dasatinib, attenuate RTEC senescence by activating SIRT1, which subsequently initiates PINK1/Parkin-mediated mitophagy (Liu et al., 2020; Wang and Wu, 2025). Resveratrol is a multifunctional phenolic compound found in various natural plants. Studies have shown that resveratrol can reduce serum AGE levels and reverse the p53/p21-and C/EBP homologous protein (CHOP)-mediated cellular senescence pathways activated by AGEs (Lan et al., 2023). Resveratrol can also inhibit renal tubular apoptosis, alleviate oxidative stress, and improve renal fibrosis by activating SIRT1 and downregulating the NOX4/ROS pathway (Uddin et al., 2021). Quercetin and resveratrol can also reduce methylglyoxal (MG) levels in DKD cells and upregulate the expression of the anti-senescence marker protein senescence marker protein 30 (SMP30), thereby reversing high-glucose-induced cellular senescence; when used in combination, the two compounds exhibit a superior synergistic effect in reducing MG levels and increasing SMP30 expression (Abharzanjani and Hemmati, 2021). However, quercetin alone has limited ability to clear senescent cells and must rely on the synergistic effect of dasatinib; the use of dasatinib is associated with rare renal adverse reactions (Adegbite et al., 2023). Moreover, natural polyphenols like resveratrol are plagued by poor aqueous solubility and extensive first-pass metabolism, resulting in suboptimal systemic bioavailability. Overcoming these pharmacokinetic limitations will necessitate structural optimization or the development of advanced drug delivery systems.

## 5 Conclusion and perspectives

RTEC senescence plays a fundamental role in the pathogenesis of DKD, serving as a core driver of tubulointerstitial fibrosis and progressive renal dysfunction. In recent years, although significant progress has been made in elucidating the mechanisms of senescence—including metabolic dysregulation, ROS and DNA damage, paracrine effects, epigenetic regulation, and the failure of endogenous protective mechanisms—clinical targeted interventions for senescent RTECs still face significant challenges. Although existing treatments such as SGLT2 inhibitors and metformin can indirectly improve metabolism, they lack direct molecular targets, making it difficult to precisely eliminate senescent cells. MSCs and their EVs still require further evaluation regarding their ability to achieve strict renal tropism and long-term safety. Meanwhile, D+Q carries a potential risk of nephrotoxicity, while quercetin and resveratrol alone suffer from low bioavailability.

In the future, in-depth research into the dynamic crosstalk and synergistic pathogenic mechanisms between RTEC senescence and stress events in the renal tubular microenvironment will be key to broadening the scope of senotherapeutic targets. At the same time, there is an urgent need to develop a new generation of anti-senescence drug delivery systems that combine multi-target regulation capabilities with low toxicity and tissue selectivity. By developing targeted carriers capable of specifically recognizing surface markers on senescent RTECs, it is expected that the local accumulation efficiency and bioavailability of anti-senescence agents in the renal tubules will be significantly enhanced, thereby maximizing therapeutic efficacy while minimizing the risk of systemic off-target effects. Finally, establishing standardized preclinical-to-clinical translational pipelines—including the generation of humanized mouse models or organoid systems that recapitulate DKD-associated senescence, the identification of reliable senescence biomarkers for patient stratification, the design of rational combination regimens, and the conduct of large-scale clinical trials—will be critical to steadily advancing anti-senescence strategies for DKD from bench to bedside.

## References

[B1] Abbad L. Esteve E. Chatziantoniou C. (2025). Advances and challenges in kidney fibrosis therapeutics. Nat. Rev. Nephrol. 21 (5), 314–329. 10.1038/s41581-025-00934-5 39934355

[B2] Abharzanjani F. Hemmati M. (2021). Protective effects of quercetin and resveratrol on aging markers in kidney under high glucose condition: *in vivo* and *in vitro* analysis. Mol. Biology Reports 48 (7), 5435–5442. 10.1007/s11033-021-06550-3 34273031

[B3] Acquah-Mills J. Henneh I. T. Arhin S. M. Ekor M. (2025). The prevalence, pattern of use and impact of traditional, complementary and alternative medicine (TCAM) among patients with end stage renal disease undergoing haemodialysis at a tertiary health facility in Ghana. Clin. Tradit. Med. Pharmacol. 6 (3), 200232. 10.1016/j.ctmp.2025.200232

[B4] Adegbite B. O. Abramson M. H. Gutgarts V. Musteata F. M. Chauhan K. Muwonge A. N. (2023). Patient-specific pharmacokinetics and dasatinib nephrotoxicity. Clin. J. Am. Soc. Nephrol. 18 (9), 1175–1185. 10.2215/CJN.0000000000000219 PMC10564352 37382967

[B5] Ahmad M. Abramovich I. Agranovich B. Nemirovski A. Gottlieb E. Hinden L. (2022). Kidney proximal tubule GLUT2-More than meets the eye. Cells 12 (1), 94. 10.3390/cells12010094 PMC9818791 36611887

[B6] Ajibade T. O. Oliyide E. O. Esan O. O. Adetona M. O. Awoyomi O. V. Oyagbemi T. O. (2024). Protective effects of naringin on fipronil-induced cardiovascular and renal dysfunctions in rats. Clin. Tradit. Med. Pharmacol. 5 (2), 200138. 10.1016/j.ctmp.2024.200138

[B7] Al-Dabet M. M. Shahzad K. Elwakiel A. Sulaj A. Kopf S. Bock F. (2022). Reversal of the renal hyperglycemic memory in diabetic kidney disease by targeting sustained tubular p21 expression. Nat. Communications 13 (1), 5062. 10.1038/s41467-022-32477-9 PMC9420151 36030260

[B8] Amarasiri S. S. Attanayake A. P. Mudduwa L. K. B. Jayatilaka K. A. P. W. (2025). Toxicity assessment of a novel polyherbal formulation with promising nephroprotective effects. Clin. Tradit. Med. Pharmacol. 6 (1), 200191. 10.1016/j.ctmp.2024.200191

[B9] Anders H. J. Huber T. B. Isermann B. Schiffer M. (2018). CKD in diabetes: diabetic kidney disease versus nondiabetic kidney disease. Nat. Rev. Nephrol. 14 (6), 361–377. 10.1038/s41581-018-0001-y 29654297

[B10] Bai Y. Chi K. Zhao D. Shen W. Liu R. Hao J. (2024). Identification of functional heterogeneity of immune cells and tubular-immune cellular interplay action in diabetic kidney disease. J. Transl. Intern. Med. 12(4), 395–405. 10.2478/jtim-2023-0130 PMC11444470 39360161

[B11] Balakumar P. (2024). Unleashing the pathological role of epithelial-to-mesenchymal transition in diabetic nephropathy: the intricate connection with multifaceted mechanism. World J. Nephrol. 13 (2), 95410. 10.5527/wjn.v13.i2.95410 38983761 PMC11229833

[B12] Barrera-Chimal J. Lima-Posada I. Bakris G. L. Jaisser F. (2022). Mineralocorticoid receptor antagonists in diabetic kidney disease - mechanistic and therapeutic effects. Nat. Rev. Nephrol. 18 (1), 56–70. 10.1038/s41581-021-00490-8 34675379

[B13] Békés M. Langley D. R. Crews C. M. (2022). PROTAC targeted protein degraders: the past is prologue. Nat. Reviews. Drug Discovery 21 (3), 181–200. 10.1038/s41573-021-00371-6 PMC8765495 35042991

[B14] Ben-Azu B. Asiwe J. N. Oritsemuelebi B. Chidebe E. O. Onuelu J. E. Isibor H. (2025). Morin inhibits oxidative stress and inflammation in the cardiorenal system associated with post-traumatic stress and alcohol use disorders in mice. Clin. Tradit. Med. Pharmacol. 6 (1), 200196. 10.1016/j.ctmp.2025.200196

[B15] Bian X. Snow Z. K. Zinn C. J. Bratulin A. L. Elhusseiny K. M. Tchkonia T. (2026). Senolytics, dasatanib plus quercetin, reduce kidney inflammation, senescent cell abundance, and injury while restoring geroprotective factors in murine diabetic kidney disease. EBioMedicine 124, 106124. 10.1016/j.ebiom.2026.106124 PMC12857402 41564845

[B16] Cai T. Ke Q. Fang Y. Wen P. Chen H. Yuan Q. (2020). Sodium-glucose cotransporter 2 inhibition suppresses HIF-1α-mediated metabolic switch from lipid oxidation to glycolysis in kidney tubule cells of diabetic mice. Cell Death and Disease 11 (5), 390. 10.1038/s41419-020-2544-7 32444604 PMC7242894

[B17] Cao G. Miao H. Wang Y. N. Chen D. Q. Wu X. Q. Chen L. (2022). Intrarenal 1-methoxypyrene, an aryl hydrocarbon receptor agonist, mediates progressive tubulointerstitial fibrosis in mice. Acta Pharmacol. Sin. 43 (11), 2929–2945. 10.1038/s41401-022-00914-6 PMC9622813 35577910

[B18] Cao J. Dun Z. Tian D. Qiao Z. Wang Y. (2026). Metabolic crosstalk in diabetic kidney disease: synergistic effects of glucotoxicity and lipotoxicity. Diabetes, Metabolic Syndrome Obesity Targets Therapy 19, 580866. 10.2147/dmso.s580866 PMC13023403 41907101

[B19] Chen K. Dai H. Yuan J. Chen J. Lin L. Zhang W. (2018). Optineurin-mediated mitophagy protects renal tubular epithelial cells against accelerated senescence in diabetic nephropathy. Cell Death Dis. 9 (2), 105. 10.1038/s41419-017-0127-z 29367621 PMC5833650

[B20] Chen K. Chen J. Wang L. Yang J. Xiao F. Wang X. (2020). Parkin ubiquitinates GATA4 and attenuates the GATA4/GAS1 signaling and detrimental effects on diabetic nephropathy. FASEB Journal Official Publication Fed. Am. Soc. Exp. Biol. 34 (7), 8858–8875. 10.1096/fj.202000053R 32436607

[B21] Chen J. Chen K. H. Wang L. M. Luo J. Zheng Q. Y. He Y. N. (2022). Decoy receptor 2 mediates the apoptosis-resistant phenotype of senescent renal tubular cells and accelerates renal fibrosis in diabetic nephropathy. Cell Death and Disease 13 (6), 522. 10.1038/s41419-022-04972-w PMC9166763 35661704

[B22] Chen B. H. Lu X. Q. Liang X. H. Wang P. (2023). Serpin E1 mediates the induction of renal tubular degeneration and premature senescence upon diabetic insult. Sci. Reports 13 (1), 16210. 10.1038/s41598-023-43411-4 PMC10533493 37758806

[B23] Chen L. Gao C. Yin X. Mo L. Cheng X. Chen H. (2024a). Partial reduction of interleukin-33 signaling improves senescence and renal injury in diabetic nephropathy. MedComm 5 (11), e742. 10.1002/mco2.742 PMC11502718 39465143

[B24] Chen Y. Chen M. Zhu W. Zhang Y. Liu P. Li P. (2024b). Morroniside attenuates podocytes lipid deposition in diabetic nephropathy: a network pharmacology, molecular docking and experimental validation study. Int. Immunopharmacology 138, 112560. 10.1016/j.intimp.2024.112560 38959541

[B25] Chen Z. Ye L. Zhu M. Xia C. Fan J. Chen H. (2024c). Single cell multi-omics of fibrotic kidney reveal epigenetic regulation of antioxidation and apoptosis within proximal tubule. Cell Mol. Life Sci. 81 (1), 56. 10.1007/s00018-024-05118-1 PMC10811088 38270638

[B26] Chen J. Fang R. Huang Q. Zhang B. Ren Z. Tan X. (2025a). Empagliflozin slows Down natural kidney senescence via Six1/Wnt4/NF-κB pathway. Biogerontology 26 (6), 193. 10.1007/s10522-025-10328-6 41109894

[B27] Chen J. He J. Wang X. Bai L. Yang X. Chen J. (2025b). Glis1 inhibits RTEC cellular senescence and renal fibrosis by downregulating histone lactylation in DKD. Life Sciences 361, 123293. 10.1016/j.lfs.2024.123293 39643036

[B28] Chen Y. Min J. Wu M. Yang R. Yu D. (2025c). Therapeutic effect of the compound *Centella asiatica* in patients with stages 4-5 CKD and its influence on serum α-Klotho and FGF23 levels. Clin. Tradit. Med. Pharmacol. 6 (1), 200202. 10.1016/j.ctmp.2025.200202

[B29] Chu M. Jiang S. Xue J. Li W. Jing G. Li H. (2025). Premature renal epithelial cell senescence promoted by LXN/Rps3/p53 signaling pathway activation increases calcium oxalate crystal deposition by altering macrophage polarization. Front. Immunology 16, 1658989. 10.3389/fimmu.2025.1658989 PMC12527873 41112268

[B30] Corradi V. Caprara C. Barzon E. Mattarollo C. Zanetti F. Ferrari F. (2024). A possible role of p-cresyl sulfate and indoxyl sulfate as biomarkers in the prediction of renal function according to the GFR (G) categories. Integr. Med. Nephrol. Androl. 11 (3), e2400002. 10.1097/imna-d-24-00002

[B31] Correa-Rotter R. Maple-Brown L. J. Sahay R. Tuttle K. R. Ulasi I. I. (2024). New and emerging therapies for diabetic kidney disease. Nat. Rev. Nephrol. 20 (3), 156–160. 10.1038/s41581-023-00782-1 38168661

[B32] Dai Y. Wu C. Zheng J. Zhao K. Hua S. Sun J. (2026). Recalibrating cell fate: targeting the mitochondrial signaling hub with natural active compounds to inhibit regulated cell death in diabetic kidney disease. Front. Physiology 17, 1774714. 10.3389/fphys.2026.1774714 PMC13050704 41948367

[B33] De Gregorio V. Barua M. Lennon R. (2025). Collagen formation, function and role in kidney disease. Nat. Rev. Nephrol. 21 (3), 200–215. 10.1038/s41581-024-00902-5 39548215

[B34] Deng Y. Zhu H. Xing J. Gao J. Duan J. Liu P. (2025). The role of natural products in improving lipid metabolism disorder-induced mitochondrial dysfunction of diabetic kidney disease. Front. Physiology 16, 1624077. 10.3389/fphys.2025.1624077 PMC12234459 40630391

[B35] Ding H. Zhou Y. Zhu R. H. Yu S. Zhang A. Q. Liu H. (2025). HIF-1-mediated macrophage metabolic reprogramming promotes AKI to CKD transition. Int. J. Biol. Sci. 21 (13), 5936–5955. 10.7150/ijbs.111238 PMC12509917 41079928

[B36] Disease G. C. K. Collaboration (2020). Global, regional, and national burden of chronic kidney disease, 1990-2017: a systematic analysis for the global burden of disease study 2017. Lancet London, Engl. 395 (10225), 709–733. 10.1016/s0140-6736(20)30045-3 PMC7049905 32061315

[B37] Dong X. Zhang J. Li W. Li Y. Jia L. Liu Z. (2024). Yi-shen-hua-shi regulates intestinal microbiota dysbiosis and protects against proteinuria in patients with chronic kidney disease: a randomized controlled study. Pharm. Biol. 62 (1), 356–366. 10.1080/13880209.2024.2345080 PMC11085992 38720666

[B38] Eleftheriadis T. Pissas G. Filippidis G. Efthymiadi M. Liakopoulos V. Stefanidis I. (2022). Dapagliflozin prevents high-glucose-induced cellular senescence in renal tubular epithelial cells. Int. Journal Molecular Sciences 23 (24), 16107. 10.3390/ijms232416107 PMC9781434 36555751

[B39] Elwakiel A. Gupta D. Rana R. Manoharan J. Al-Dabet M. M. Ambreen S. (2024). Factor XII signaling via uPAR-integrin β1 axis promotes tubular senescence in diabetic kidney disease. Nat. Communications 15 (1), 7963. 10.1038/s41467-024-52214-8 PMC11390906 39261453

[B40] Feng Q. Su C. Yang C. Wu M. Li X. Lin X. (2024). RXRα/MR signaling promotes diabetic kidney disease by facilitating renal tubular epithelial cells senescence and metabolic reprogramming. Transl. Research The Journal Laboratory Clinical Medicine 274, 101–117. 10.1016/j.trsl.2024.10.001 39424127

[B41] Fioretto P. Pontremoli R. (2022). Expanding the therapy options for diabetic kidney disease. Nat. Rev. Nephrol. 18 (2), 78–79. 10.1038/s41581-021-00522-3 34862514

[B42] Gao P. Zou X. Sun X. Zhang C. (2022). Cellular senescence in metabolic-associated kidney disease: an update. Cells 11 (21), 3443. 10.3390/cells11213443 PMC9657665 36359836

[B43] Gao X. Chen L. Zhang C. Gao H. Yang S. Huang G. (2025). Total *Panax notoginseng* saponins treats diabetic kidney disease in mice by regulating the TXNIP/NLRP3 signaling pathway. Clin. Tradit. Med. Pharmacol. 6 (4), 200245. 10.1016/j.ctmp.2025.200245

[B44] Glastras S. J. Pollock C. A. (2024). Targeted identification of risk and treatment of diabetic kidney disease. Nat. Rev. Nephrol. 20 (2), 75–76. 10.1038/s41581-023-00796-9 38040863

[B45] Göbel K. Eichler S. Wiendl H. Chavakis T. Kleinschnitz C. Meuth S. G. (2018). The coagulation factors fibrinogen, thrombin, and factor XII in inflammatory Disorders-A systematic review. Front. Immunol. 9, 1731. 10.3389/fimmu.2018.01731 30105021 PMC6077258

[B46] Gu L. Liu B. Zhang L. Zhao J. He W. Lang H. (2026). SGLT2 inhibition attenuates renal tubular senescence by suppressing CTRP1-Mediated glucotoxic stress in diabetic kidney disease. FASEB Journal Official Publication Fed. Am. Soc. Exp. Biol. 40 (14), e72142. 10.1096/fj.202601959R PMC13374599 42461151

[B47] Han X. Liao R. Li X. Zhang C. Huo S. Qin L. (2025). Mesenchymal stem cells in treating human diseases: molecular mechanisms and clinical studies. Signal Transduction Targeted Therapy 10 (1), 262. 10.1038/s41392-025-02313-9 PMC12371117 40841367

[B48] He M. Cao C. Ni Z. Liu Y. Song P. Hao S. (2022). PROTACs: great opportunities for academia and industry (an update from 2020 to 2021). Signal Transduction Targeted Therapy 7 (1), 181. 10.1038/s41392-022-00999-9 PMC9178337 35680848

[B49] He H. Wang H. Chen X. Zhong Y. Huang X. R. Ma R. C. (2024). Treatment for type 2 diabetes and diabetic nephropathy by targeting Smad3 signaling. Int. J. Biol. Sci. 20 (1), 200–217. 10.7150/ijbs.87820 PMC10750285 38164169

[B50] He X. Guo Y. Zhao Y. (2025). Targeting gut microbiota as a promising therapy for chronic kidney disease. Integr. Med. Nephrol. Androl. 12 (3), e25–e26. 10.1097/imna-d-25-00026

[B51] Hickson L. J. Eirin A. Conley S. M. Taner T. Bian X. Saad A. (2021). Diabetic kidney disease alters the transcriptome and function of human adipose-derived mesenchymal stromal cells but maintains immunomodulatory and paracrine activities important for renal repair. Diabetes 70 (7), 1561–1574. 10.2337/db19-1268 PMC8336004 33858824

[B52] Hong Q. Kim H. Cai G. Y. Chen X. M. He J. C. Lee K. (2025). Modulation of TGF-β signaling new approaches toward kidney disease and fibrosis therapy. Int. J. Biol. Sci. 21 (4), 1649–1665. 10.7150/ijbs.101548 PMC11844295 39990662

[B53] Hou Y. Tan E. Shi H. Ren X. Wan X. Wu W. (2024). Mitochondrial oxidative damage reprograms lipid metabolism of renal tubular epithelial cells in the diabetic kidney. Cell Mol. Life Sci. 81 (1), 23. 10.1007/s00018-023-05078-y PMC10781825 38200266

[B54] Hu M. Hu H. Hu J. Chen Z. Yang K. Zhang Z. (2026a). ERRα-NOS2-mediated citrulline metabolism attenuates tubular epithelial cell senescence in diabetic kidney disease. Redox Biology 90, 104065. 10.1016/j.redox.2026.104065 PMC12906104 41643324

[B55] Hu M. Lv L. Li R. Zhao Z. Li W. Wang Y. (2026b). NAT10 inhibition alleviates renal tubular epithelial cell senescence by impeding ac4C acetylation of PAPP-A mRNA in diabetic nephropathy. Cell Death Dis. 17 (1), 738. 10.1038/s41419-026-08970-0 PMC13478519 42276981

[B56] Huang W. Hickson L. J. Eirin A. Kirkland J. L. Lerman L. O. (2022). Cellular senescence: the good, the bad and the unknown. Nat. Reviews. Nephrol. 18 (10), 611–627. 10.1038/s41581-022-00601-z PMC9362342 35922662

[B57] Jia J. Tan R. Xu L. Wang H. Li J. Su H. (2024). Hederagenin improves renal fibrosis in diabetic nephropathy by regulating Smad3/NOX4/SLC7A11 signaling-mediated tubular cell ferroptosis. Int. Immunopharmacology 135, 112303. 10.1016/j.intimp.2024.112303 38776855

[B58] Jiang X. Ruan X. L. Xue Y. X. Yang S. Shi M. Wang L. N. (2020). Metformin reduces the senescence of renal tubular epithelial cells in diabetic nephropathy via the MBNL1/miR-130a-3p/STAT3 pathway. Oxidative Medicine Cellular Longevity 2020, 8708236. 10.1155/2020/8708236 32104542 PMC7035567

[B59] Jiang C. H. Zhang S. J. Li P. Miao H. Zhao Y. Y. (2025). Natural products targeting TGF-β/Smad signaling in renal fibrosis: multiomics-Based novel molecular mechanisms and therapeutic strategies. Phytomedicine 148, 157496. 10.1016/j.phymed.2025.157496 41202381

[B60] Kajimoto E. Nagasu H. Takasu M. Kishi S. Wada M. Tatsugawa R. (2024). ASK-1 activation exacerbates kidney dysfunction via increment of glomerular permeability and accelerates cellular aging in diabetic kidney disease model mice. Sci. Reports 14 (1), 26438. 10.1038/s41598-024-77577-2 PMC11531550 39488570

[B61] Kato M. Natarajan R. (2019). Epigenetics and epigenomics in diabetic kidney disease and metabolic memory. Nat. Rev. Nephrol. 15 (6), 327–345. 10.1038/s41581-019-0135-6 30894700 PMC6889804

[B62] Kim B. M. Yang S. B. Ha S. Kim M. J. Kim J. Park D. E. (2026). Nanoplastic exposure induces mitochondrial dysfunction-driven inflammation and senescence in renal tubular epithelial cells. Free Radic. Biol. Med. 253, 18–35. 10.1016/j.freeradbiomed.2026.05.298 42162843

[B63] Kluge K. E. Seljeflot I. Arnesen H. Jensen T. Halvorsen S. Helseth R. (2022). Coagulation factors XI and XII as possible targets for anticoagulant therapy. Thromb. Res. 214, 53–62. 10.1016/j.thromres.2022.04.013 35490644

[B64] Ko M. S. Yun J. Y. Kim S. Kim M. O. Go S. H. Jin H. J. (2025). Mesenchymal stem cells mediated suppression of GREM2 inhibits renal epithelial-mesenchymal transition and attenuates the progression of diabetic kidney disease. Int. Journal Stem Cells 18 (2), 158–172. 10.15283/ijsc24113 PMC12122246 39757007

[B65] Konari N. Nagaishi K. Kikuchi S. Fujimiya M. (2019). Mitochondria transfer from mesenchymal stem cells structurally and functionally repairs renal proximal tubular epithelial cells in diabetic nephropathy *in vivo* . Sci. Reports 9 (1), 5184. 10.1038/s41598-019-40163-y 30914727 PMC6435708

[B66] Lai W. Wang B. Huang R. Zhang C. Fu P. Ma L. (2024). Ferroptosis in organ fibrosis: from mechanisms to therapeutic medicines. J. Transl. Intern. Med. 12(1), 22–34. 10.2478/jtim-2023-0137 PMC10956731 38525436

[B67] Lan K. C. Peng P. J. Chang T. Y. Liu S. H. (2023). Resveratrol alleviates advanced glycation end-products-related renal dysfunction in D-Galactose-Induced aging mice. Metabolites 13 (5), 655. 10.3390/metabo13050655 37233696 PMC10224182

[B68] Li S. Susztak K. (2025). Mitochondrial dysfunction has a central role in diabetic kidney disease. Nat. Rev. Nephrol. 21 (2), 77–78. 10.1038/s41581-024-00919-w 39681602

[B69] Li S. Livingston M. J. Ma Z. Hu X. Wen L. Ding H. F. (2023a). Tubular cell senescence promotes maladaptive kidney repair and chronic kidney disease after cisplatin nephrotoxicity. JCI Insight 8 (8), e166643. 10.1172/jci.insight.166643 PMC10243740 36917180

[B70] Li X. Guo L. Chen J. Liang H. Liu Y. Chen W. (2023b). Intravenous injection of human umbilical cord-derived mesenchymal stem cells ameliorates not only blood glucose but also nephrotic complication of diabetic rats through autophagy-mediated anti-senescent mechanism. Stem Cell Research and Therapy 14 (1), 146. 10.1186/s13287-023-03354-z PMC10228071 37248536

[B71] Li X. Li C. Zhang W. Wang Y. Qian P. Huang H. (2023c). Inflammation and aging: signaling pathways and intervention therapies. Signal Transduction Targeted Therapy 8 (1), 239. 10.1038/s41392-023-01502-8 PMC10248351 37291105

[B72] Li Q. Liu J. Su R. Zhen J. Liu X. Liu G. (2024a). Small extracellular vesicles-shuttled miR-23a-3p from mesenchymal stem cells alleviate renal fibrosis and inflammation by inhibiting KLF3/STAT3 axis in diabetic kidney disease. Int. Immunopharmacology 139, 112667. 10.1016/j.intimp.2024.112667 39018690

[B73] Li X. J. Fang C. Zhao R. H. Zou L. Miao H. Zhao Y. Y. (2024b). Bile acid metabolism in health and ageing-related diseases. Biochem. Pharmacol. 225, 116313. 10.1016/j.bcp.2024.116313 38788963

[B74] Li X. J. Suo P. Wang Y. N. Zou L. Nie X. L. Zhao Y. Y. (2024c). Arachidonic acid metabolism as a therapeutic target in AKI-to-CKD transition. Front. Pharmacol. 15, 1365802. 10.3389/fphar.2024.1365802 PMC10957658 38523633

[B75] Li X. J. Wang Y. N. Wang W. F. Nie X. Miao H. Zhao Y. Y. (2024d). Barleriside A, an aryl hydrocarbon receptor antagonist, ameliorates podocyte injury through inhibiting oxidative stress and inflammation. Front. Pharmacol. 15, 1386604. 10.3389/fphar.2024.1386604 PMC11374728 39239643

[B76] Li Y. Min X. Zhang X. Cao X. Kong Q. Mao Q. (2024e). HSPA12A promotes c-Myc lactylation-mediated proliferation of tubular epithelial cells to facilitate renal functional recovery from kidney ischemia/reperfusion injury. Cell Mol. Life Sci. 81 (1), 404. 10.1007/s00018-024-05427-5 PMC11402889 39277835

[B77] Li Z. Wang X. Tasich K. Hike D. Schumacher J. G. Zhou Q. (2025). Eupatilin unveiled: an in-depth exploration of research advancements and clinical therapeutic prospects. J. Translational Internal Medicine 13 (2), 104–117. 10.1515/jtim-2025-0016 PMC12116271 40443403

[B78] Liang D. Li Z. Feng Z. Yuan Z. Dai Y. Wu X. (2022). Metformin improves the senescence of renal tubular epithelial cells in a high-glucose state through E2F1. Front. Pharmacology 13, 926211. 10.3389/fphar.2022.926211 PMC9262145 35814218

[B79] Liao C. M. Luo T. von der Ohe J. de Juan Mora B. Schmitt R. Hass R. (2021). Human MSC-derived exosomes reduce cellular senescence in renal epithelial cells. Int. Journal Molecular Sciences 22 (24), 13562. 10.3390/ijms222413562 PMC8709122 34948355

[B80] Liles J. T. Corkey B. K. Notte G. T. Budas G. R. Lansdon E. B. Hinojosa-Kirschenbaum F. (2018). ASK1 contributes to fibrosis and dysfunction in models of kidney disease. J. Clinical Investigation 128 (10), 4485–4500. 10.1172/jci99768 30024858 PMC6159961

[B81] Lin D. W. Yang T. M. Ho C. Shih Y. H. Lin C. L. Hsu Y. C. (2024). Targeting macrophages: therapeutic approaches in diabetic kidney disease. Int. Journal Molecular Sciences 25 (8), 4350. 10.3390/ijms25084350 PMC11050450 38673935

[B82] Liu Y. (2024). Kidney fibrosis: fundamental questions, challenges, and perspectives. Integr. Med. Nephrol. Androl. 11 (4), e24–e27. 10.1097/imna-d-24-00027

[B83] Liu T. Yang Q. Zhang X. Qin R. Shan W. Zhang H. (2020). Quercetin alleviates kidney fibrosis by reducing renal tubular epithelial cell senescence through the SIRT1/PINK1/mitophagy axis. Life Sci. 257, 118116. 10.1016/j.lfs.2020.118116 32702447

[B84] Liu H. J. Miao H. Yang J. Z. Liu F. Cao G. Zhao Y. Y. (2023). Deciphering the role of lipoproteins and lipid metabolic alterations in ageing and ageing-associated renal fibrosis. Ageing Res. Rev. 85, 101861. 10.1016/j.arr.2023.101861 36693450

[B85] Liu D. Chen X. He W. Lu M. Li Q. Zhang S. (2024a). Update on the pathogenesis, diagnosis, and treatment of diabetic tubulopathy. Integr. Med. Nephrol. Androl. 11 (4), e23–e29. 10.1097/IMNA-D-23-00029

[B86] Liu H. Wang J. Yue G. Xu J. (2024b). Placenta-derived mesenchymal stem cells protect against diabetic kidney disease by upregulating autophagy-mediated SIRT1/FOXO1 pathway. Ren. Failure 46 (1), 2303396. 10.1080/0886022x.2024.2303396 PMC10798286 38234193

[B87] Liu P. Y. Hong K. F. Liu Y. D. Sun Z. Y. Zhao T. T. Li X. L. (2024c). Total flavonoids of astragalus protects glomerular filtration barrier in diabetic kidney disease. Chin. Med. 19 (1), 27. 10.1186/s13020-024-00903-3 PMC10870499 38365794

[B88] Liu H. Xiang X. Shi C. Guo J. Ran T. Lin J. (2025a). Oxidative stress and inflammation in renal fibrosis: novel molecular mechanisms and therapeutic targets. Chem. Biol. Interact. 421, 111784. 10.1016/j.cbi.2025.111784 41110496

[B89] Liu K. Yu M. He Y. Li Y. Huang X. R. Li G. (2025b). Smad3 mediates renal fibrosis via GPX4-Dependent ferroptosis. Int. J. Biol. Sci. 21 (13), 5922–5935. 10.7150/ijbs.114075 41079940 PMC12509913

[B90] Liu X. Chen J. Gu S. Chen Y. Zhang R. Zang Q. (2025c). Prolonged glucagon exposure rewires lipid oxidation and drives diabetic kidney disease progression. Nat. Communications 16 (1), 8561. 10.1038/s41467-025-63529-5 PMC12479795 41022721

[B91] Liu X. Zhang C. Fu Y. Xie L. Kong Y. Yang X. (2025d). Inflammation, apoptosis, and fibrosis in diabetic nephropathy: molecular crosstalk in proximal tubular epithelial cells and therapeutic implications. Curr. Issues Molecular Biology 47 (11), 885. 10.3390/cimb47110885 PMC12651756 41296389

[B92] Liu L. Yu Q. Q. Zhang Y. L. Zhou J. T. Jin Y. Jiang C. H. (2026a). Renal fibrosis is induced by hyperactive Wnt/β-catenin pathway via microbial-mediated tryptophan metabolism-driven AhR signaling in rodents and humans. Cell Mol. Life Sci. 83 (1), 182. 10.1007/s00018-026-06176-3 PMC13038810 41862737

[B93] Liu L. M. Fang H. B. Wang Y. F. Zhang Y. L. Yu Q. Q. Zhang W. Y. (2026b). Niaoduqing particles ameliorated tubulointerstitial fibrosis by suppressing IκB/NF-κB signalling pathway via inhibiting host- and gut microbiota-mediated tryptophan co-metabolism. Microbiol. Res. 311, 128592. 10.1016/j.micres.2026.128592 42335503

[B94] Liu L. M. He X. F. Zhang Y. L. Zhou J. T. Miao H. Zhao Y. Y. (2026c). Gut microbiota and renal fibrosis: novel mechanistic insights and therapeutic potential. Acta Pharmacol. Sin. 47 (8), 2023–2034. 10.1038/s41401-026-01801-0 PMC13389047 41951771

[B95] Liu L. M. Zhang Y. L. Zhou J. T. Yu Q. Q. Zhang W. Y. Wang W. F. (2026d). Ureic clearance granule ameliorates chronic kidney disease by reshaping microbial dysbiosis via modulating bile acid metabolism. Chin. Med. 21 (1), 155. 10.1186/s13020-026-01423-y PMC13220541 42216070

[B96] Long Y. Song D. Xiao L. Xiang Y. Li D. Sun X. (2024). m(6)A RNA methylation drives kidney fibrosis by upregulating β-catenin signaling. Int. J. Biol. Sci. 20 (8), 3185–3200. 10.7150/ijbs.96233 38904026 PMC11186362

[B97] Lu J. Li X. Q. Chen P. P. Zhang J. X. Li L. Wang G. H. (2024a). Acetyl-CoA synthetase 2 promotes diabetic renal tubular injury in mice by rewiring fatty acid metabolism through SIRT1/ChREBP pathway. Acta Pharmacol. Sin. 45 (2), 366–377. 10.1038/s41401-023-01160-0 PMC10789804 37770579

[B98] Lu J. Sun W. Liu B. Zhang J. Wang R. Goltzman D. (2024b). Chk2 modulates bmi1-deficiency-induced renal aging and fibrosis via oxidative stress, DNA damage, and p53/TGF81-induced epithelial-mesenchymal transition. Int. J. Biol. Sci. 20 (6), 2008–2026. 10.7150/ijbs.93598 PMC11008269 38617548

[B99] Lu X. Wu J. Agborbesong E. Li X. (2025). P16-positive senescent cells promote DKD by the dysregulation of glycolysis and mitochondrial metabolism. Cell Death Discovery 11 (1), 355. 10.1038/s41420-025-02650-2 40738954 PMC12311014

[B100] Lukášová E. Kovařík A. Kozubek S. (2018). Consequences of lamin B1 and lamin B receptor downregulation in senescence. Cells 7 (2), 11. 10.3390/cells7020011 29415520 PMC5850099

[B101] Luo Y. Zhang L. Su N. Liu L. Zhao T. (2024). YME1L-mediated mitophagy protects renal tubular cells against cellular senescence under diabetic conditions. Biol. Research 57 (1), 10. 10.1186/s40659-024-00487-0 PMC10946153 38494498

[B102] Maas C. Renné T. (2018). Coagulation factor XII in thrombosis and inflammation. Blood 131 (17), 1903–1909. 10.1182/blood-2017-04-569111 29483100

[B103] Mao W. Yang N. Zhang L. Li C. Wu Y. Ouyang W. (2020). Bupi yishen formula versus losartan for non-diabetic stage 4 chronic kidney disease: a randomized controlled trial. Front. Pharmacol. 11, 627185. 10.3389/fphar.2020.627185 33708125 PMC7941267

[B104] Mei R. Wan Z. Yang C. Shen X. Wang R. Zhang H. (2024). Advances and clinical challenges of mesenchymal stem cell therapy. Front. Immunology 15, 1421854. 10.3389/fimmu.2024.1421854 PMC11294097 39100671

[B105] Miao H. Vaziri N. D. (2025). Hederagenin: molecular mechanisms and therapeutic potential for chronic kidney disease. Integr. Med. Nephrol. Androl. 12(3)**,** e2500017. 10.1097/IMNA-D-25-00017

[B106] Miao H. Zhang Y. M. Yu X. Y. Zou L. Zhao Y. Y. (2022). Membranous nephropathy: systems biology-based novel mechanism and traditional Chinese medicine therapy. Front. Pharmacol. 13, 969930. 10.3389/fphar.2022.969930 36176440 PMC9513429

[B107] Miao H. Wang Y. N. Su W. Zou L. Zhuang S. G. Yu X. Y. (2023). Sirtuin 6 protects against podocyte injury by blocking the renin-angiotensin system by inhibiting the Wnt1/β-catenin pathway. Acta Pharmacol. Sin. 45 (1), 137–149. 10.1038/s41401-023-01148-w 37640899 PMC10770168

[B108] Miao H. Wang K. E. Li P. Zhao Y. Y. (2025). Rhubarb: traditional uses, phytochemistry, multiomics-based novel pharmacological and toxicological mechanisms. Drug Des. Devel Ther. 19, 9457–9480. 10.2147/dddt.s557114 PMC12558105 41158982

[B109] Ni Y. Chen Y. Jiang X. Pu T. Zhang L. Li S. (2023). Transplantation of human amniotic mesenchymal stem cells Up-Regulates angiogenic factor expression to attenuate diabetic kidney disease in rats. Diabetes, Metabolic Syndrome Obesity Targets Therapy 16, 331–343. 10.2147/dmso.s371752 PMC9921454 36785675

[B110] Nikfarjam S. Rezaie J. Zolbanin N. M. Jafari R. (2020). Mesenchymal stem cell derived-exosomes: a modern approach in translational medicine. J. Translational Medicine 18 (1), 449. 10.1186/s12967-020-02622-3 33246476 PMC7691969

[B111] Pan D. Qu Y. Shi C. Xu C. Zhang J. Du H. (2024). Oleanolic acid and its analogues: promising therapeutics for kidney disease. Chin. Med. 19 (1), 74. 10.1186/s13020-024-00934-w 38816880 PMC11140902

[B112] Patel H. A. Wang J. Zinn C. J. Learmonth M. Bian X. Jurk D. (2026). Targeting cellular senescence in diabetic kidney disease: potential of regenerative, cell-based therapies and other senotherapeutic approaches. Kidney Int. 109 (4), 684–703. 10.1016/j.kint.2025.10.019 41581730 PMC13523092

[B113] Pham M. Stoll G. Nieswandt B. Bendszus M. Kleinschnitz C. (2012). Blood coagulation factor XII--a neglected player in stroke pathophysiology. J. Mol. Med. Berl. 90 (2), 119–126. 10.1007/s00109-011-0812-9 21909687

[B114] Phillips P. C. A. de Sousa Loreto Aresta Branco M. Cliff C. L. Ward J. K. Squires P. E. Hills C. E. (2025). Targeting senescence to prevent diabetic kidney disease: exploring molecular mechanisms and potential therapeutic targets for disease management. Diabet. Medicine A Journal Br. Diabet. Assoc. 42 (2), e15408. 10.1111/dme.15408 PMC11733669 38995865

[B115] Qi R. Yang C. (2018). Renal tubular epithelial cells: the neglected mediator of tubulointerstitial fibrosis after injury. Cell Death Dis. 9 (11), 1126. 10.1038/s41419-018-1157-x 30425237 PMC6233178

[B116] Qiao P. Zhu Y. Zhang J. Gan Y. Wang Y. Li Y. (2026). Targeting DYRK1A with harmine abrogates the FOXO1-NF-κB axis to alleviate cellular senescence and renal fibrosis. Mech. Ageing Dev. 231, 112184. 10.1016/j.mad.2026.112184 42119856

[B117] Rayego-Mateos S. Rodrigues-Diez R. R. Fernandez-Fernandez B. Mora-Fernández C. Marchant V. Donate-Correa J. (2023). Targeting inflammation to treat diabetic kidney disease: the road to 2030. Kidney Int. 103 (2), 282–296. 10.1016/j.kint.2022.10.030 36470394

[B118] Robbins P. D. Jurk D. Khosla S. Kirkland J. L. LeBrasseur N. K. Miller J. D. (2021). Senolytic drugs: reducing senescent cell viability to extend health span. Annu. Review Pharmacology Toxicology 61, 779–803. 10.1146/annurev-pharmtox-050120-105018 32997601 PMC7790861

[B119] Ruan Z. Liu J. Liu W. Huang W. (2024). Qufeng tongluo decoction May alleviate podocyte injury induced by high glucose and hydrogen peroxide by regulating autophagy. Integr. Med. Nephrol. Androl. 11 (4), e2400023. 10.1097/IMNA-D-24-00023

[B120] Ruan Y. Wang D. Xu Y. Yang J. Chen Y. Fan J. (2026). VHL-Recruiting PROTAC attenuates AKI-CKD transition via simultaneous degradation of Smad3 and stabilization of HIF-2α. Cell Death and Disease 17 (1), 460. 10.1038/s41419-026-08726-w 41963313 PMC13181044

[B121] Schunk S. J. Floege J. Fliser D. Speer T. (2021). WNT-β-catenin signalling - a versatile player in kidney injury and repair. Nat. Rev. Nephrol. 17 (3), 172–184. 10.1038/s41581-020-00343-w 32989282

[B122] Scisciola L. Olivieri F. Ambrosino C. Barbieri M. Rizzo M. R. Paolisso G. (2023). On the wake of metformin: do anti-diabetic SGLT2 inhibitors exert anti-aging effects? Ageing Research Reviews 92, 102131. 10.1016/j.arr.2023.102131 37984626

[B123] Shamanaev A. Litvak M. Ivanov I. Srivastava P. Sun M.-F. Dickeson S. (2023). Factor XII structure–function relationships. Seminars Thrombosis Hemostasis 50, 937–952. 10.1055/s-0043-1769509 PMC10696136 37276883

[B124] Sharma K. Hansen J. Susztak K. Eberlin L. Anderton C. R. Alexandrov T. (2025). Spatial metabolomics and multiomics integration for breakthroughs in precision medicine for kidney disease. Nat. Rev. Nephrol. 22 (2), 152–164. 10.1038/s41581-025-01007-3 41068470 PMC13264805

[B125] Small D. M. Bennett N. C. Roy S. Gabrielli B. G. Johnson D. W. Gobe G. C. (2012). Oxidative stress and cell senescence combine to cause maximal renal tubular epithelial cell dysfunction and loss in an *in vitro* model of kidney disease. Nephron Exp. Nephrol. 122 (3-4), 123–130. 10.1159/000350726 23735887

[B126] Song Y. J. Yang B. Feng Q. S. Ma F. F. Xing B. Bin X. L. (2025). Gut microbiota-derived trimethylamine N-oxide exacerbates diabetic nephropathy by promoting renal fibrosis. World J. Nephrol. 14 (4), 112066. 10.5527/wjn.v14.i4.112066 PMC12754428 41479812

[B127] Stepanova N. (2024). Probiotic interventions in peritoneal dialysis: a review of underlying mechanisms and therapeutic potentials. World J. Nephrol. 13 (4), 98719. 10.5527/wjn.v13.i4.98719 39723354 PMC11572655

[B128] Sun J. Yin C. Li Z. Gao X. Li S. Gao H. (2025). Natural compounds regulating fatty acid oxidation in the treatment of diabetic kidney disease. Front. Nutrition 12, 1669557. 10.3389/fnut.2025.1669557 PMC12568393 41170356

[B129] Sun Z. H. Gong Q. Wang Z. L. Li C. Wang J. N. Yu J. T. (2026). Microbiota-gut-kidney axis and targeted therapeutic strategies in kidney diseases. Int. J. Biol. Sci. 22 (3), 1142–1161. 10.7150/ijbs.124499 41608641 PMC12836503

[B130] Tang S. C. W. Yiu W. H. (2020). Innate immunity in diabetic kidney disease. Nat. Rev. Nephrol. 16 (4), 206–222. 10.1038/s41581-019-0234-4 31942046

[B131] Teh Y. M. Mualif S. A. Mohd Noh N. I. Lim S. K. (2024). The potential of naturally derived compounds for treating chronic kidney disease: a review of autophagy and cellular senescence. Int. J. Mol. Sci. 26 (1), 3. 10.3390/ijms26010003 39795863 PMC11719669

[B132] Tesch G. H. Ma F. Y. Nikolic-Paterson D. J. (2016). ASK1: a new therapeutic target for kidney disease. Am. Journal Physiology. Ren. Physiology 311 (2), F373–F381. 10.1152/ajprenal.00208.2016 27226108

[B133] Tesch G. H. Ma F. Y. Ozols E. Nikolic-Paterson D. J. (2024). Intervention treatment reducing cellular senescence inhibits tubulointerstitial fibrosis in diabetic mice following acute kidney injury. Clin. Science Lond. Engl. 1979 138 (5), 309–326. 10.1042/cs20231698 PMC10914710 38391050

[B134] Tian Y. Chen J. Huang W. Ren Q. Feng J. Liao J. (2024). Myeloid-derived wnts play an indispensable role in macrophage and fibroblast activation and kidney fibrosis. Int. J. Biol. Sci. 20 (6), 2310–2322. 10.7150/ijbs.94166 38617540 PMC11008274

[B135] Trigo C. M. Rodrigues J. S. Camões S. P. Solá S. Miranda J. P. (2025). Mesenchymal stem cell secretome for regenerative medicine: where do we stand? J. Advanced Research 70, 103–124. 10.1016/j.jare.2024.05.004 PMC11976416 38729561

[B136] Uddin M. J. Farjana M. Moni A. Hossain K. S. Hannan M. A. Ha H. (2021). Prospective pharmacological potential of resveratrol in delaying kidney aging. Int. Journal Molecular Sciences 22 (15), 8258. 10.3390/ijms22158258 PMC8348580 34361023

[B137] Ueda S. Tominaga T. Ochi A. Sakurai A. Nishimura K. Shibata E. (2021). TGF-β1 is involved in senescence-related pathways in glomerular endothelial cells via p16 translocation and p21 induction. Sci. Reports 11 (1), 21643. 10.1038/s41598-021-01150-4 PMC8569175 34737348

[B138] Walther C. P. Winkelmayer W. C. Richardson P. A. Virani S. S. Navaneethan S. D. (2021). Renin-angiotensin system blocker discontinuation and adverse outcomes in chronic kidney disease. Nephrol. Dial. Transpl. 36 (10), 1893–1899. 10.1093/ndt/gfaa300 PMC8633426 33367872

[B139] Wang W. Wu W. (2025). Therapeutic effects of quercetin on renal fibrosis and injury. Integr. Med. Nephrol. Androl. 12 (1). 10.1097/IMNA-D-24-00051

[B140] Wang Z. Han N. Zhao K. Li Y. Chi Y. Wang B. (2019). Protective effects of pyrroloquinoline quinine against oxidative stress-induced cellular senescence and inflammation in human renal tubular epithelial cells via Keap1/Nrf2 signaling pathway. Int. Immunopharmacol. 72, 445–453. 10.1016/j.intimp.2019.04.040 31035086

[B141] Wang D. Yin L. Chen R. Tan W. Liang L. Xiang J. (2022). Senescent renal tubular epithelial cells activate fibroblasts by secreting shh to promote the progression of diabetic kidney disease. Front. Medicine 9, 1018298. 10.3389/fmed.2022.1018298 PMC9905119 36760880

[B142] Wang Y. N. Miao H. Yu X. Y. Guo Y. Su W. Liu F. (2023a). Oxidative stress and inflammation are mediated via aryl hydrocarbon receptor signalling in idiopathic membranous nephropathy. Free Radic. Biol. Med. 207, 89–106. 10.1016/j.freeradbiomed.2023.07.014 37451370

[B143] Wang Y. N. Zhang Z. H. Liu H. J. Guo Z. Y. Zou L. Zhang Y. M. (2023b). Integrative phosphatidylcholine metabolism through phospholipase A_2_ in rats with chronic kidney disease. Acta Pharmacol. Sin. 44 (2), 393–405. 10.1038/s41401-022-00947-x PMC9889763 35922553

[B144] Wang J. Liu H. Yue G. Deng Y. Cai W. Xu J. (2024a). Human placenta-derived mesenchymal stem cells ameliorate diabetic kidney disease by modulating the T helper 17 cell/regulatory T-cell balance through the programmed death 1/programmed death-ligand 1 pathway. Diabetes, Obesity and Metabolism 26 (1), 32–45. 10.1111/dom.15282 37722965

[B145] Wang J. Wang J. Lu C. Wang Y. Bi H. Zheng J. (2024b). ISL1-overexpressing BMSCs attenuate renal ischemia-reperfusion injury by suppressing apoptosis and oxidative stress through the paracrine action. Cell Mol. Life Sci. 81 (1), 312. 10.1007/s00018-024-05354-5 PMC11335236 39066917

[B146] Wang T. Huang Y. Zhang X. Zhang Y. Zhang X. (2024c). Advances in metabolic reprogramming of renal tubular epithelial cells in sepsis-associated acute kidney injury. Front. Physiol. 15, 1329644. 10.3389/fphys.2024.1329644 PMC10834781 38312312

[B147] Wang W. Dai R. Cheng M. Chen Y. Gao Y. Hong X. (2024d). Metabolic reprogramming and renal fibrosis: what role might Chinese medicine play? Chin. Med. 19 (1), 148. 10.1186/s13020-024-01004-x PMC11514863 39465434

[B148] Wang Y. Liu T. Wu Y. Wang L. Ding S. Hou B. (2024e). Lipid homeostasis in diabetic kidney disease. Int. J. Biol. Sci. 20 (10), 3710–3724. 10.7150/ijbs.95216 PMC11302873 39113692

[B149] Wang Y. N. Li X. J. Wang W. F. Zou L. Miao H. Zhao Y. Y. (2024f). Geniposidic acid attenuates chronic tubulointerstitial nephropathy through regulation of the NF‐ƙB/Nrf2 pathway via aryl hydrocarbon receptor signaling. Phytother. Res. 38 (11), 5441–5457. 10.1002/ptr.8324 39289784

[B150] Wang C. Chen D. Yin Y. Liu X. Liu Y. Li X. (2025a). Multi-target regulation of cellular senescence by traditional Chinese medicine: a novel strategy to preventing diabetic kidney disease. Ren. Failure 47 (1), 2511275. 10.1080/0886022x.2025.2511275 PMC12893495 40538167

[B151] Wang Y. Shao M. Li T. Du X. Chen K. Xu L. (2025b). Xian huang Fang inhibits the PKCδ/MAPK signaling pathway to attenuate renal tubular epithelial cell senescence and thereby delay renal interstitial fibrosis. Drug Des. Devel Ther. 19, 6991–7004. 10.2147/dddt.s522318 PMC12358136 40827228

[B152] Wang Y. B. Li T. Wang F. Y. Yao X. Bai Q. X. Su H. W. (2025c). The dual role of cellular senescence in macrophages: unveiling the hidden driver of age-related inflammation in kidney disease. Int. J. Biol. Sci. 21 (2), 632–657. 10.7150/ijbs.104404 PMC11705649 39781471

[B153] Wang K. E. Yu Q. Q. Wang W. F. Wang Y. N. Yang J. Liu L. M. (2026a). Natural product therapy in diabetic kidney disease: emerging multiomics-mediated signalling pathway and molecular target. Chin. Med. 21 (1), 1511. 10.1186/s13020-026-01473-2 PMC13599269 42778934

[B154] Wang W. Zhu H. Zhao K. Wang X. Liang Y. Xu J. (2026b). Natural products modulate renal metabolic reprogramming in diabetic kidney disease. J. Transl. Intern. Med. 14(4), 524–546. 10.1515/jtim-2026-0050 PMC13509154 42657136

[B155] Wei Y. Mou S. Yang Q. Liu F. Cooper M. E. Chai Z. (2024). To target cellular senescence in diabetic kidney disease: the known and the unknown. Clin. Science Lond. Engl. 1979 138 (16), 991–1007. 10.1042/cs20240717 PMC11327223 39139135

[B156] Wei W. Yang L. Wang B. Tang L. Li J. Liu C. (2025). Remote ischemic preconditioning attenuates mitochondrial dysfunction and ferroptosis of tubular epithelial cells by inhibiting NOX4-ROS signaling in acute kidney injury. Int. J. Biol. Sci. 21 (5), 2313–2329. 10.7150/ijbs.105667 PMC11900797 40083709

[B157] Wu W. Wang Y. Shao X. Huang S. Wang J. Zhou S. (2024). GLP-1RA improves diabetic renal injury by alleviating glomerular endothelial cells pyrotosis via RXRα/circ8411/miR-23a-5p/ABCA1 pathway. PloS One 19 (12), e0314628. 10.1371/journal.pone.0314628 PMC11611192 39621727

[B158] Wu X. Sun W. Xing L. Zhao J. (2025). Effect of yishenqinglihuoxue formula in the treatment of patients with stage 5 chronic kidney disease using propensity score matching: a retrospective clinical study. Clin. Tradit. Med. Pharmacol. 6 (3), 200239. 10.1016/j.ctmp.2025.200239

[B159] Wu G. Chen F. Xiong Q. (2026). Bidirectional regulation between mitochondrial metabolic reprogramming and epigenetic modifications in renal tubular epithelial cell injury of diabetic kidney disease. Front. Endocrinol. (Lausanne) 17, 1831426. 10.3389/fendo.2026.1831426 PMC13471077 42598512

[B160] Xiang H. L. Yuan Q. Zeng J. Y. Xu Z. Y. Zhang H. Z. Huang J. (2024). MDM2 accelerated renal senescence via ubiquitination and degradation of HDAC1. Acta Pharmacol. Sin. 45 (11), 2328–2338. 10.1038/s41401-024-01294-9 PMC11489730 38760541

[B161] Xiao M. Tang D. Luan S. Hu B. Gong W. Pommer W. (2023). Dysregulated coagulation system links to inflammation in diabetic kidney disease. Front. Clinical Diabetes Healthcare 4, 1270028. 10.3389/fcdhc.2023.1270028 PMC10748384 38143793

[B162] Xiong Y. B. Huang W. Y. Ling X. Zhou S. Wang X. X. Li X. L. (2024). Mitochondrial calcium uniporter promotes kidney aging in mice through inducing mitochondrial calcium-mediated renal tubular cell senescence. Acta Pharmacologica Sin. 45 (10), 2149–2162. 10.1038/s41401-024-01298-5 PMC11420221 38789496

[B163] Yan Z. Xu J. Liu T. Wang L. Zhang Q. Li X. (2025a). The role of renal cell senescence in diabetic kidney disease: mechanisms and therapeutic advances. Diabetes Metab. Syndr. Obes. 18, 3323–3341. 10.2147/dmso.s543814 PMC12428652 40949087

[B164] Yan Z. Zhang L. Ma T. Yuan Y. Kang Y. Liu S. (2025b). SuoquanYishen formula improves renal cellular senescence by inhibiting YTHDF1-Rubicon axis to promote autophagy in diabetic kidney disease. Front. Pharmacol. 16, 1543277. 10.3389/fphar.2025.1543277 PMC12075247 40371338

[B165] Yang C. Chen X. C. Li Z. H. Wu H. L. Jing K. P. Huang X. R. (2021). SMAD3 promotes autophagy dysregulation by triggering lysosome depletion in tubular epithelial cells in diabetic nephropathy. Autophagy 17 (9), 2325–2344. 10.1080/15548627.2020.1824694 33043774 PMC8496726

[B166] Yang Y. Mihajlovic M. Janssen M. J. Masereeuw R. (2023). The uremic toxin indoxyl sulfate accelerates senescence in kidney proximal tubule cells. Toxins (Basel) 15 (4), 242. 10.3390/toxins15040242 PMC10143766 37104179

[B167] Yang M. Liu Y. Luo S. L. Liu C. B. Jiang N. Li C. R. (2024a). DsbA-L ameliorates renal aging and renal fibrosis by maintaining mitochondrial homeostasis. Acta Pharmacol. Sin. 45 (4), 777–789. 10.1038/s41401-023-01216-1 PMC10943083 38200148

[B168] Yang M. Wu S. Dai Q. Qin W. Zhang Y. Lei Y. (2024b). Andrographolide prevents renal fibrosis via decelerating lipotoxicity-mediated premature senescence of tubular epithelial cells. Biochem. Pharmacology 230 (Pt 3), 116615. 10.1016/j.bcp.2024.116615 39528075

[B169] Yang M. Li J. Huang X. Jin S. Wan S. Wu S. (2025). AT1, a small molecular degrader of BRD4 based on proteolysis targeting chimera technology alleviates renal fibrosis and inflammation in diabetic nephropathy. Bioorg. Chem. 156, 108184. 10.1016/j.bioorg.2025.108184 39862737

[B170] Yang W. X. Pang Y. C. Liao M. C. Peng J. Leal D. N. Yamashita M. (2026). Intrarenal renin-angiotensin system inactivation ameliorates tubulopathy via attenuation of mitochondrial oxidative stress, dysfunction and senescence in diabetes. Clin. Science Lond. Engl. 1979 140 (3), 321–338. 10.1042/cs20258557 41657319

[B171] Yin L. Tian F. Li Z. Chen W. Liu C. Zhang Y. (2025). Benzo[b]fluoranthene is involved in idiopathic membranous nephropathy by inducing podocyte injury. J. Transl. Intern. Med. 13(2), 118–127. 10.1515/jtim-2025-0022 PMC12116268 40443401

[B172] Yu D. Luo L. Liang Y. Zhou H. Xiao Y. An X. (2025). ER-α36 prevents high glucose-induced cellular senescence and apoptosis in renal tubular cell. Front. Endocrinology 16, 1426854. 10.3389/fendo.2025.1426854 PMC12183064 40551891

[B173] Yuan T. Gao X. Wang X. Tang X. Liang Z. Yin Y. (2025). Yiqi wenyang formula ameliorates diabetic kidney disease via inhibiting inflammation and regulating the gut microbiota-bile acid axis in mice by FXR signaling pathway. Chin. Med. 20 (1), 183. 10.1186/s13020-025-01238-3 PMC12574177 41168780

[B174] Zhang Q. Qi J. Luo Q. Wu M. Zhang L. Qin L. (2022). Yishen xiezhuo formula ameliorates the development of cisplatin-induced acute kidney injury by attenuating renal tubular epithelial cell senescence. Ann. Transl. Med. 10 (24), 1392. 10.21037/atm-22-5415 PMC9843381 36660714

[B175] Zhang H. F. Liu H. M. Xiang J. Y. Zhou X. C. Wang D. Chen R. Y. (2023). Alpha lipoamide inhibits diabetic kidney fibrosis via improving mitochondrial function and regulating RXRα expression and activation. Acta Pharmacologica Sin. 44 (5), 1051–1065. 10.1038/s41401-022-00997-1 PMC10104876 36347997

[B176] Zhang K. Zheng S. Wu J. He J. Ouyang Y. Ao C. (2024). Human umbilical cord mesenchymal stem cell-derived exosomes ameliorate renal fibrosis in diabetic nephropathy by targeting Hedgehog/SMO signaling. FASEB Journal Official Publication Fed. Am. Soc. Exp. Biol. 38 (7), e23599. 10.1096/fj.202302324R 38572590

[B177] Zhang Y. Qi X. Sun R. X. Gong Y. F. Yang H. K. Wang W. L. (2025a). Dendrobine ameliorates mitophagy-mediated endothelial senescence in diabetic kidney disease through activating the SIRT1/FOXO3a pathway. Chin. Med. 20 (1), 176. 10.1186/s13020-025-01234-7 PMC12534925 41107952

[B178] Zhang Y. Sun Z. Meng W. Lei S. Sun J. Tang Y. (2025b). Celastrol attenuates diabetic kidney disease progression by repressing senescence of renal tubular epithelial cells. Front. Aging 6, 1657947. 10.3389/fragi.2025.1657947 PMC12696166 41393102

[B179] Zhang W. Y. Zhang Y. L. Yu Q. Q. Zhou J. T. Miao H. Liu L. M. (2026). Gut microbial-derived metabolites: key players in kidney disease and renal fibrosis. Int. J. Biol. Sci. 22 (10), 5421–5443. 10.7150/ijbs.133969 PMC13215364 42212329

[B180] Zhao H. Ma L. Yan M. Wang Y. Zhao T. Zhang H. (2018). Association between MYH9 and APOL1 gene polymorphisms and the risk of diabetic kidney disease in patients with type 2 diabetes in a Chinese Han population. J. Diabetes Research 2018, 5068578. 10.1155/2018/5068578 29862302 PMC5971321

[B181] Zhao L. Hu C. Zhang P. Jiang H. Chen J. (2019). Preconditioning strategies for improving the survival rate and paracrine ability of mesenchymal stem cells in acute kidney injury. J. Cell Mol. Med. 23 (2), 720–730. 10.1111/jcmm.14035 30484934 PMC6349184

[B182] Zhao L. Hao Y. Tang S. Han X. Li R. Zhou X. (2023a). Energy metabolic reprogramming regulates programmed cell death of renal tubular epithelial cells and might serve as a new therapeutic target for acute kidney injury. Front. Cell Dev. Biol. 11, 1276217. 10.3389/fcell.2023.1276217 PMC10694365 38054182

[B183] Zhao X. P. Chang S. Y. Pang Y. Liao M. C. Peng J. Ingelfinger J. R. (2023b). Hedgehog interacting protein activates sodium-glucose cotransporter 2 expression and promotes renal tubular epithelial cell senescence in a mouse model of type 1 diabetes. Diabetologia 66 (1), 223–240. 10.1007/s00125-022-05810-6 36260124

[B184] Zhao B. R. Hu X. R. Wang W. D. Zhou Y. (2025). Cardiorenal syndrome: clinical diagnosis, molecular mechanisms and therapeutic strategies. Acta Pharmacol. Sin. 46 (6), 1539–1555. 10.1038/s41401-025-01476-z PMC12098865 39910210

[B185] Zhong Q. Zeng J. Li Y. Zhang H. Lin T. Song T. (2025). Senescent renal tubular cells derived extracellular vesicles transported miR-20a and miR-21 induced macrophage-to-myofibroblast transition in renal fibrosis after ischemia reperfusion injury. Int. J. Biol. Sci. 21 (3), 940–954. 10.7150/ijbs.97579 PMC11781180 39897045

[B186] Zhou S. Lan Q. Xin W. Wang Y. Zhang A. Xiong J. (2025). Targeting NINJ1-mediated plasma membrane rupture in tubular epithelial cell prevents inflammatory response in acute kidney injury. Int. J. Biol. Sci. 21 (12), 5223–5239. 10.7150/ijbs.115364 PMC12435343 40959284

[B187] Zhu H. Sun J. Yan Y. Liu P. Huang Y. (2026). Immune podocyte injury in autoimmune glomerular diseases. Front. Immunology 17, 1804416. 10.3389/fimmu.2026.1804416 41939877 PMC13043406

